# The Role of Salicylic, Jasmonic Acid and Ethylene in the Development of the Resistance/Susceptibility of Wheat to the SnTox1-Producing Isolate of the Pathogenic Fungus *Stagonospora nodorum* (Berk.)

**DOI:** 10.3390/plants13182546

**Published:** 2024-09-10

**Authors:** Svetlana Veselova, Tatyana Nuzhnaya, Igor Maksimov

**Affiliations:** 1 Institute of Biochemistry and Genetics, Ufa Federal Research Centre, Russian Academy of Sciences, Prospekt Oktyabrya, 71, 450054 Ufa, Russia; tanyawww89@mail.ru (T.N.); maksimov@ufaras.ru (I.M.); 2 Ufa Institute of Biology, Ufa Federal Research Centre, Russian Academy of Sciences, Prospekt Oktyabrya, 69, 450054 Ufa, Russia

**Keywords:** catalase, fungal pathogen, hormone crosstalk, hydrogen peroxide, necrotrophic effectors, phytohormones, plant–microbe interaction, SnTox1, *Stagonospora nodorum*, transcription factors

## Abstract

The SnTox1 effector is a virulence factor of the fungal pathogen *Stagonospora nodorum* (Berk.), which interacts with the host susceptibility gene *Snn*1 in a gene-for-gene manner and causes necrosis on the leaves of sensitive wheat genotypes. It is known that salicylic acid (SA), jasmonic acid (JA) and ethylene are the key phytohormones involved in plant immunity. To date, effectors of various pathogens have been discovered that can manipulate plant hormonal pathways and even use hormone crosstalk to promote disease development. However, the role of SnTox1 in manipulating hormonal pathways has not been studied in detail. We studied the redox status and the expression of twelve genes of hormonal pathways and two MAPK genes in six bread wheat cultivars sensitive and insensitive to SnTox1 with or without treatment by SA, JA and ethephon (ethylene-releasing agent) during infection with the SnTox1-producing isolate *S. nodorum* 1SP. The results showed that SnTox1 controls the antagonism between the SA and JA/ethylene signaling pathways. The SA pathway was involved in the development of susceptibility, and the JA/ethylene pathways were involved in the development of wheat plants resistance to the Sn1SP isolate in the presence of a SnTox1-*Snn1* interaction. SnTox1 hijacked the SA pathway to suppress catalase activity, increase hydrogen peroxide content and induce necrosis formation; it simultaneously suppresses the JA and ethylene hormonal pathways by SA. To do this, SnTox1 reprogrammed the expression of the MAPK genes *TaMRK3* and *TaMRK6* and the TF genes *TaWRKY13*, *TaEIN3* and *TaWRKY53b*. This study provides new data on the role of SnTox1 in manipulating hormonal pathways and on the role of SA, JA and ethylene in the pathosystem wheat *S. nodorum*.

## 1. Introduction

Plant pathogens annually cause serious yield losses in many crops, including wheat, so studying the mechanisms of the development of plant defense reactions is an urgent task. According to modern concepts, plant immune reactions in response to pathogen attack are induced at several levels [1]. The first level of defense is the nonspecific recognition of the elicitor molecules of pathogens, which leads to the development of basal immunity, known as PAMP (pathogen-associated molecular patterns)-triggered immunity or pattern-triggered immunity (PTI) [1]. The second level of defense is the specific recognition of pathogen effectors by the products of effector-specific plant genes, called effector-triggered immunity (ETI) and corresponds to a specific gene-for-gene response [1]. ETI results in the limitation of the pathogen growth and development of the resistance when biotrophic pathogens attack. Effectors of necrotrophic pathogens (NEs) interact with the products of dominant susceptibility genes (*S*-genes) and cause infection; in addition, they suppress PTI and use the host’s ETI pathway to develop susceptibility, resulting in NE-triggered susceptibility (NETS), and this corresponds to the inverse gene-for-gene interaction [2]. Recent studies have reported various interactions between two immune systems—nonspecific (PTI) and specific (ETI), but the mechanism of these relationships is not fully understood [3,4]. However, it is known that the induction of PTI and ETI causes similar reactions in plants: ion-flux changes, mitogen-activated protein kinase (MAPK) cascade activation, the regulation of the generation of reactive oxygen species (ROS), synthesis lignin, callose deposition, the activation of the transcription of pathogenesis-related genes (PR genes), etc., and these responses can be influenced by pathogen effectors [1,4].

According to their mode of nutrition—biotrophy, necrotrophy or hemibiotrophy—pathogens have evolved different infection strategies and, therefore, secrete different effectors. Biotrophic effectors suppress immunity and minimize damage to host cells [5]. Necrotrophic effectors cause the necrosis of host plant cells. Hemibiotrophic pathogens selectively secrete different effectors at different spatial and temporal levels. They suppress the basal immunity of the plant during the biotrophic phase and cause cell death during the necrotrophic phase of development [5,6]. In addition, the action of effectors is aimed at destroying physical barriers and creating favorable conditions to growth and development, masking and protecting the pathogen and interfering with the hormonal signaling systems of the host plant [7]. Phytohormones play a key regulatory role in plant immune responses. Salicylic acid (SA), jasmonic acid (JA) and ethylene are classical plant hormones of immunity and constitute a central regulatory network that interacts with other phytohormones such as cytokinins (CKs), auxins, abscisic acid (ABA), brassinosteroids (BR) and gibberellins (Gas) [8,9]. It is known that SA-induced resistance is directed against biotrophic pathogens [6,8]. Meanwhile, ethylene together with JA forms plant resistance to necrotrophic pathogens and pests [6,8]. Effectors usually affect the biosynthesis, metabolism and/or signaling pathway of phytohormone, and their target can be transcription factors (TFs)—modulators and regulators of hormonal signaling pathways [3,6]. Transcription factor ERF121, from the AP2/ERF family, was identified as a target gene for the TAL effector (transcription activator-like effector (TALE)) *Xanthomonas campestris* [10]. It was assumed that the TALE-dependent activation of ERF121 promotes susceptibility to *X. campestris* by hijacking the ethylene signaling pathway [10].

Typically, phytohormones do not act individually but through complex antagonistic or synergistic interactions, and this network is often called the hormone crosstalk [11]. The antagonism of SA and JA/ethylene, manifested in the development of protective reactions against biotrophic and necrotrophic pathogens, and the synergism of JA and ethylene in relation to protection against necrotrophic pathogens and pests are well known [11]. Hormone crosstalk is thought to provide benefits to plants by allowing the simultaneous regulation of different processes, resulting in an increased ability to respond to different types of stresses [11]. However, some pathogens are able to use hormone crosstalk to promote disease development [5]. The XopS effector of *X. campestris* stabilizes the negative regulator of the SA signaling pathway WRKY40, which leads to a decrease in the expression of its target SA response genes, and activates the JA pathway by suppressing the JA repressor JAZ8, which promotes successful plant colonization [12]. Most of the compelling evidence for these processes has been obtained primarily from the model plant *Arabidopsis thaliana*, while crops such as wheat remain poorly studied.

The pathogenic fungus *Stagonospora nodorum* (Berk.) is the most harmful member of the Dothideomycetes class, causing the Septoria nodorum blotch (SNB) of the spring and winter wheat [13]. The main virulence factors of the pathogen are NEs, encoded by the *SnTox* genes, the products of which interact with the products of the host-plant susceptibility genes (*Snn*), causing the development of the disease [14]. To date, about a dozen interactions have been characterized [14]. The effectors SnToxA, SnTox1 and SnTox3 are quite widespread among strains and isolates, causing necrosis and chlorosis in susceptible wheat genotypes, and their role in the suppression of PTI and the development of NETS is being actively studied [15]. This work focuses on the SnTox1-*Snn1* interaction, which plays an important role in the development of the disease [16]. The *SnTox1* gene encodes a cysteine-rich peptide with a chitin-binding motif, which causes the extension of necrosis in wheat with the *Snn1* gene located on chromosome 1BS [16,17]. The high content of cysteine residues in the SnTox1 protein indicates that NE can function in the apoplastic space of plants similar to a biotrophic effector (avirulence factor) by activating programmed cell death (PCD) [16]. It was proven that the SnTox1-*Snn1* interaction caused the induction of the MAPK cascade, increased the production of hydrogen peroxide (H_2_O_2_) and triggered the activation of the transcription of genes encoding pathogenesis-related proteins (PR-proteins) and DNA laddering, which led to the development of PCD and the formation of extensive necrosis, which provided the pathogen with nutrients and conditions for sporulation [16]. It has also been proven that SnTox1 performs another function, which is provided by its chitin-binding motif and is associated with the protection of infectious structures of the fungus from interaction with plant chitinases and destruction [16]. These results demonstrate that NE SnTox1 can hijack the host molecular pathways involved in the resistance to biotrophic pathogens, but the role of SnTox1 in manipulating hormonal signaling pathways has not been studied in detail.

We hypothesize that SnTox1 may manipulate hormonal signaling pathways using the antagonism of the SA and JA/ethylene pathways in the development of resistance responses, and this may be associated with influencing the activity of redox enzymes involved in the generation and utilization of H_2_O_2_. Previously, we showed that NE SnTox3 induces the ethylene signaling pathway, regulating ROS production in wheat at the early stage of *S. nodorum* infection. Our previous results suggest that the activation of the ethylene signaling pathway was aimed at suppression SA-dependent defense responses and PTI in wheat susceptible to *S. nodorum* isolates producing SnTox3 [13].

In this regard, the goal of our work was to identify the role of the NE SnTox1 in manipulating the hormonal signaling pathways of SA, JA and ethylene in wheat plants in order to regulate the redox status of plants and the formation of necrosis, as well as to determine the role of each of the three phytohormones in the development of the resistance/susceptibility of wheat to SnTox1-producing *S. nodorum* isolate and summarize the obtained data and make a model of the influence of SnTox1 on the hormonal signaling pathways of wheat plants. For this study, based on our previous works and the works of other authors, six cultivars of bread wheat (*Triticum aestivum* L.) with different sensitivity to NE SnTox1 (insensitive, moderately sensitive and sensitive) were selected [17,18]. To identify the role of SnTox1 in the regulation of the redox status of wheat through the manipulation of plant hormonal signaling pathways and the generation of H_2_O_2_, the activity of peroxidase (PO) and catalase (CAT) enzymes was studied in six different cultivars of bread wheat with or without treatment by phytohormones infected with the SnTox1-producing *S. nodorum* isolate. Additionally, to study the effect of NE SnTox1 on hormonal signaling pathways, we analyzed the transcriptional activity of twelve genes of the biosynthesis and signaling pathway of SA, JA and ethylene in the leaves of six different wheat cultivars with or without treatment by phytohormones infected with the SnTox1-producing *S. nodorum* isolate. Among these twelve genes, the expression of the *PR* genes, involved in the plant defense response to pathogens, including *S. nodorum* [13,14,15,16], and the expression of TF genes, being hormone crosstalk hubs and possible targets of pathogen effectors, were studied. The expression of the MAPK genes *TaMPK3* and *TaMPK6* was also studied, since some TFs are substrates for these MAPKs.

Correlation matrices were constructed that showed the positive relationship between NE SnTox1 and the generation of H_2_O_2_, the expression of SA-dependent genes, and the formation of necrosis, as well as the negative relationship with the antioxidant activity of plants and the expression of JA/ethylene-dependent genes. These results provided important information about the role of NE SnTox1 in manipulating hormonal signaling pathways and the contribution of SA, JA and ethylene to the development of wheat resistance/susceptibility to SnTox1-producing *S. nodorum* isolate.

## 2. Results

### 2.1. The Role of SnTox1 and Phytohormones SA, JA and Ethylene in the Development of Disease Symptoms in Various Wheat Genotypes

**Figure 1:**
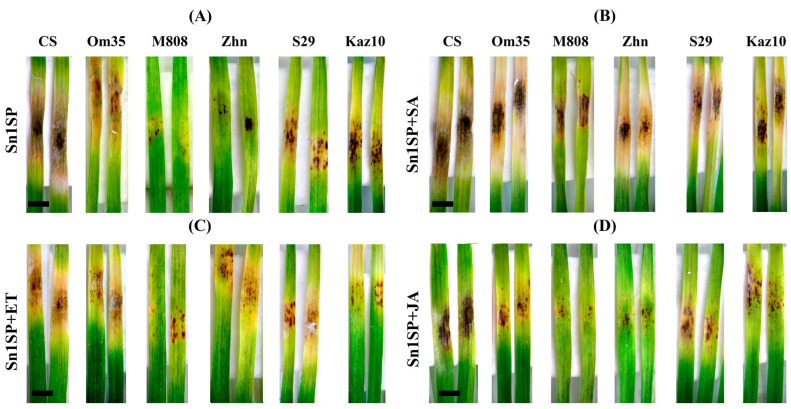
The effect of treatment with phytohormones on the development of SNB on the leaves of six cultivars of varying degrees of sensitivity to NE SnTox1 infected with the *S. nodorum* Sn1SP. Note: (**A**) wheat plants not treated with phytohormones and infected with the Sn1SP isolate (Sn1SP); (**B**) wheat plants treated with SA and infected with the Sn1SP isolate (Sn1SP + SA); (**C**) wheat plants treated with ethephon (ET) and infected with the Sn1SP isolate (Sn1SP + ET); (**D**) wheat plants treated with JA and infected with the Sn1SP isolate (Sn1SP + JA). Wheat cultivars designations: Chinese Spring (CS), Omskaya 35 (Om35), Mironovskaya808 (M808), Zhnitsa (Zhn), Saratovskaya 29 (S29) and Kazahstanskaya 10 (Kaz10). The photographs show the results of a typical variant from a series of experiments (*n* = 30). The development of symptoms was recorded 6 days after infection. Bars = 15 mm.

In this work, six cultivars were studied, two of which showed sensitivity to NE SnTox1 and susceptibility to the Sn1SP isolate. For cultivars Chinese Spring (CS) and Omskaya 35 (Om35), the leaf damage areas occupied more than 50% of the total leaf area (Table 1, Figure 1). The remaining four cultivars showed resistance to the Sn1SP isolate (insensitivity to NE SnTox1) but differed in the manifestation of symptoms. In the cultivars Mironovskaya808 (M808) and Zhnitsa (Zhn), the smallest lesion zones, which occupied only 2–5% of the total leaf area, were found (Table 1, Figure 1). In the cultivars Saratovskaya 29 (S29) and Kazahstanskaya 10 (Kaz10), the damage areas occupied approximately 11% of the total leaf area (Table 1), but the symptoms themselves were nontypical (Figure 1). In these two cultivars, many small damage zones were found that did not lead to large lesions (Figure 1); the symptoms manifested as “flecking” [19].

The results of the analysis of the influence of phytohormones on the development of SNB showed that treatment with SA led to an increase in the damage areas in both cultivars sensitive to SnTox1 (Om35) and insensitive cultivars M808, Zhn, S29 and Kaz10 (Table 1, Figure 1). Treatment with SA had little effect on the cv. CS plants and had a lesser effect on the cv. M808, S29 and Kaz10 plants than on the cv. Zhn plants, in which the damage area when treated with SA increased 15 times (Table 1, Figure 1). The treatment of plants with JA led to a reduction in damage zones and an increase in the resistance of the SnTox1-sensitive (susceptible) cv. CS and Om35 and also reduced lesions in cultivars with nontypical symptoms—S29 and Kaz10 (Table 1, Figure 1). However, the treatment of plants with JA did not affect the resistant cultivars with the smallest damage zones—M808 and Zhn (Table 1, Figure 1). The treatment of plants with ethephon (ET) had a disparate effect on the development of SNB (Table 1). In the SnTox1-sensitive cultivars CS and Om35, ET reduced the damage areas by approximately two times (Table 1, Figure 1). In the SnTox1-insensitive (resistant) cultivars M808 and S29, treatment with ET led to an increase in the damage zones by two times, and, in the ET-treated cv. Zhn plants, the affected areas increased by seven times compared to untreated and infected plants (Table 1, Figure 1). However, the affected areas did not exceed 15–18% of the total leaf area; as a result, the plants remained resistant (up to 15%) and slightly susceptible (up to 25%) according to the international disease rating scale. ET did not affect the development of the SNB of the cv. Kaz10 (Table 1, Figure 1).

**Figure 2:**
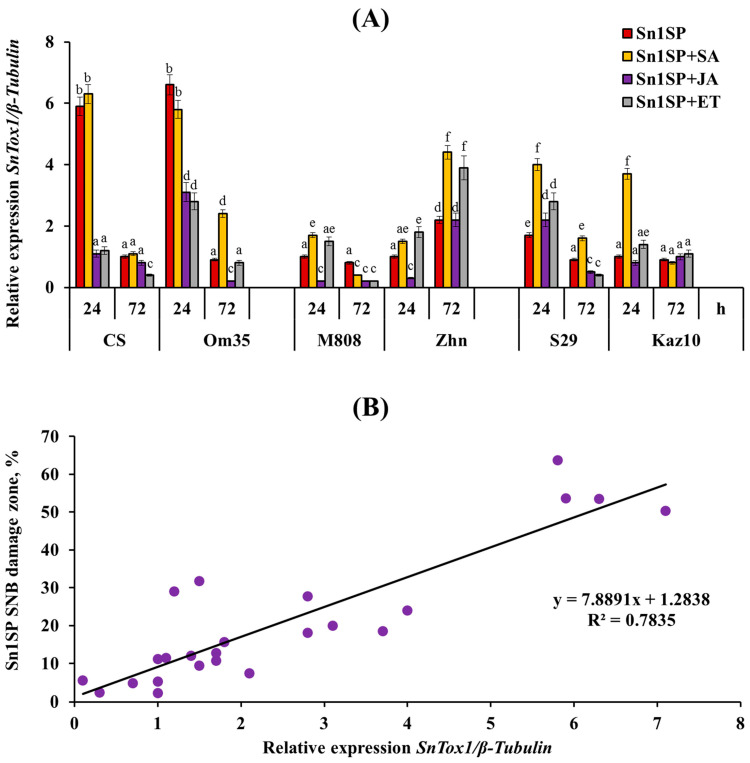
Comparative analysis of the *SnTox*1 gene expression and plant sensitivity to NE in six bread wheat cultivars during treatment with hormones and infection with Sn1SP isolate. (**A**) The expression level of the *SnTox1* gene normalized to *β-tubulin* in the six wheat cultivars treated with SA, JA and ET and infected with the Sn1SP isolate. Average gene expression was calculated from nine biological samples in five technical replicates (*n* = 9). Values are expressed as mean SE (ANOVA with Duncan’s test; comparisons between genotypes are presented; significant differences are marked with different letters at *p* ≤ 0.05). (**B**) Regression line for relationship between *SnTox1* gene expression and extent of the lesions in the six wheat cultivars treated with SA, JA and ET. The correlation coefficient of Pearson’s was *r* = 0.89, and the coefficient of determination was R^2^ = 0.78, *p*-value = 9.18 × 10^−9^.

In the current study, the relative expression of *SnTox1* to the fungal *β-tubulin* gene was examined at all infected six cultivars under all hormonal treatments using relative-quantitative PCR (Figure 2). Two susceptible cultivars, CS and Om35, showed high transcript levels of *SnTox1* gene 24 h post infection (hpi); *SnTox1* gene expression in these cultivars exceeded *β-tubulin* gene expression by six and seven times, respectively (Figure 2). In resistant cultivars, the expression of the SnTox1 gene either did not change, as in the cultivars M808 and Kaz10, or increased by approximately two times, as in the cultivars Zhn and S29, compared to the expression of the *β-tubulin* gene (Figure 2). Moreover, the expression of the *SnTox1* gene in the cv. S29 reached its peak 24 hpi, and, in the cv. Zhn, it reached 72 hpi (Figure 2).

Treatment with SA led to an increase in the mRNA abundance of the *SnTox1* gene in the SnTox1-insensitive cultivars M808, Zhn, S29 and Kaz10 24 hpi and only in three cultivars Om35, Zhn and S29 72 hpi compared to the infected, untreated plants (Figure 2). The treatment of plants with JA or ET reduced or had no effect on the expression of the *SnTox1* gene in all six cultivars 24 and 72 hpi compared to the infected, untreated plants (Figure 2).

In this work, we studied the relationship between the degree of the cultivar sensitivity to NE, which manifested itself in the development of damage zones, and the level of the expression of the *SnTox1* gene during infection and how this indicator is affected by various phytohormones. Correlation analysis showed a positive relationship between the extent of the lesions in all six wheat cultivars treated with SA, JA and ET or not and the expression level of the *SnTox1* gene 24 hpi with the Sn1SP isolate of these plants (*r* = 0.89, R^2^ = 0.78, *p*-value = 9.18 × 10^−9^) (Figure 2, Table S1, Supplementary Materials).

### 2.2. The Role of SnTox1 and Phytohormones SA, JA and Ethylene in the Regulation of the Redox Status in Various Wheat Genotypes

**Figure 3:**
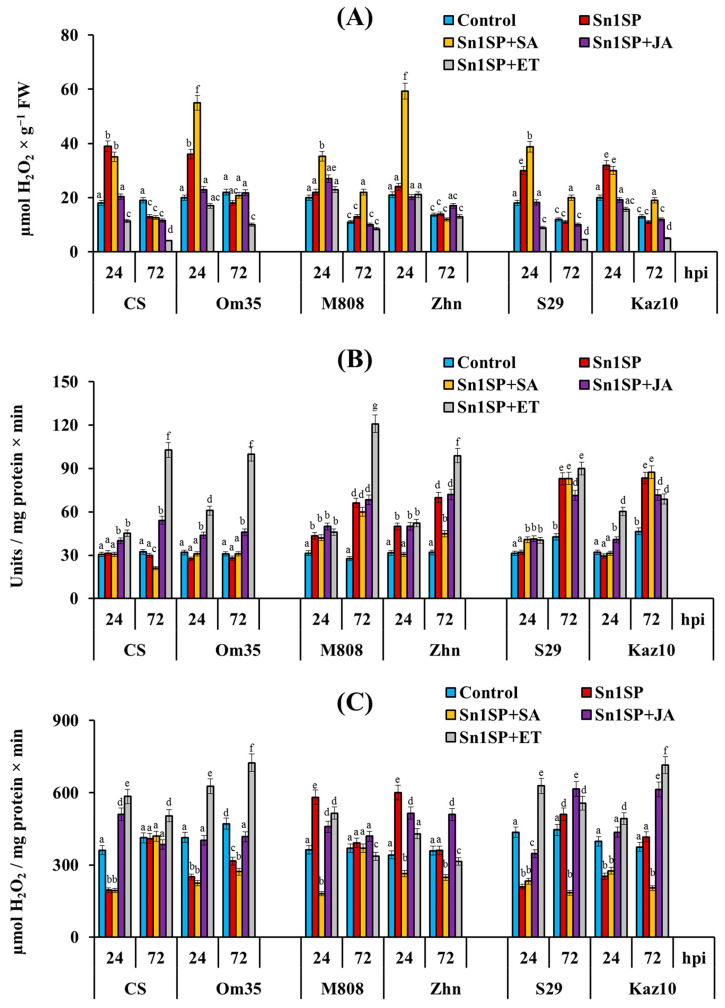
Effect of SA, JA and ET treatment on changes in H_2_O_2_ content (**A**) and activity of peroxidase (**B**) and catalase (**C**) enzymes in six cultivars of bread wheat 24 and 72 hpi with the SnTox1-producing isolate *S. nodorum* Sn1SP. Designations: control—untreated and uninfected wheat plants; Sn1SP—wheat plants untreated with phytohormones and infected with *S. nodorum* isolate Sn1SP; Sn1SP + SK—wheat plants treated with salicylic acid (SA) and infected with *S. nodorum* isolate Sn1SP; Sn1SP + JA—wheat plants treated with jasmonic acid (JA) and infected with *S. nodorum* isolate Sn1SP; Sn1SP + ET—wheat plants treated with ethephon (ET) and infected with *S. nodorum* isolate Sn1SP. Figures present means ± SE (*n* = 9). (ANOVA with Duncan’s test; comparisons between genotypes are presented; significant differences are marked with different letters at *p* ≤ 0.05.)

Analysis of the redox status of six genotypes of wheat showed that an accumulation of H_2_O_2_, an absence of an increase in PO activity and a decrease in CAT activity were detected in susceptible (SnTox1-sensitive) cultivars (CS, Om35) 24 hpi (Figure 3). The content of H_2_O_2_ did not change in the first three days of infection compared to the control, PO activity began to increase 24 hpi and greatly increased 72 hpi, and CAT activity increased by approximately two times 24 hpi and was at the control level 72 hpi in resistant (SnTox1-insensitive) cultivars (M808, Zhn) (Figure 3). Resistant cultivars with nontypical symptoms (S29 and Kaz10) showed an accumulation of H_2_O_2_, an absence of the increase in PO activity and a decrease in CAT activity 24 hpi, as in susceptible cultivars (Figure 3). However, an increase in PO activity was found in the cultivars S29 and Kaz10 72 hpi compared to the control, as in resistant cultivars (Figure 3).

The greatest increase in H_2_O_2_ content was found in SA-treated plants of the cv. Om35 and Zhn 24 hpi; with that, these cultivars showed the strongest increase in lesions during SA treatment (Table 1, Figure 3). In addition, the accumulation of H_2_O_2_ was found 72 hpi compared to the control in SA-treated resistant cultivars with nontypical symptoms (S29 and Kaz10) and cv. M808 (Figure 3). Treatment with SA reduced PO activity or did not lead to an increase in enzyme activity in susceptible cultivars (CS, Om35) and the cv. Zhn 24 and 72 hpi compared to the control (Figure 3). Also, SA treatment did not affect CAT activity in susceptible cultivars (Figure 3). However, treatment with SA greatly reduced CAT activity in the resistant cv. M808 24 hpi and in the cultivars Zhn, S29 and Kaz10 24 and 72 hpi (Figure 3). Thus, the effect of the SA treatment was aimed at reducing CAT activity to increase the content of H_2_O_2_ and the development of large lesion zones.

The treatment of plants with JA and ET affected the redox status approximately equally (Figure 3). In plants treated with JA or ET, the H_2_O_2_ content decreased or remained at the level of control plants 24 and 72 hpi (Figure 3). At the same time, treatment with JA or ET had little effect on the H_2_O_2_ content in the resistant (SnTox1-insensitive) cultivars (M808, Zhn) (Figure 3). PO activity increased in JA-treated resistant cultivars M808 and Zhn and in JA/ET-treated cultivars with nontypical symptoms (S29 and Kaz10), as well as in untreated infected plants (Figure 3). The exception was the plants of cultivars M808 and Zhn treated with ET; the activity of PO in these cultivars increased by six and three times, respectively, compared to the control 72 hpi (Figure 3). The CAT activity increased in susceptible cultivars (CS and Om35) and resistant cultivars with nontypical symptoms (S29 and Kaz10) treated with JA or ET and infected with Sn1SP (Figure 3). Treatment with ET reduced CAT activity in the cv. Zhn 24 and 72 hpi, which could cause an increase in affected areas by seven times (Table 1, Figure 3). Thus, the influence of JA and ET was aimed at reducing the content of H_2_O_2_ by increasing the activity of PO and CAT, which led to a reduction in affected areas. The only exception was the cv. Zhn.

### 2.3. The Role of SnTox1 in Manipulating the SA, JA and Ethylene Signaling Pathways and the Role of the Hormone Crosstalk in the Development of the Resistance/Susceptibility of Wheat to the Pathogen S. nodorum

#### 2.3.1. Analysis of the Expression of Twelve Genes of Hormonal Signaling Pathways in Various Wheat Genotypes

**Figure 4:**
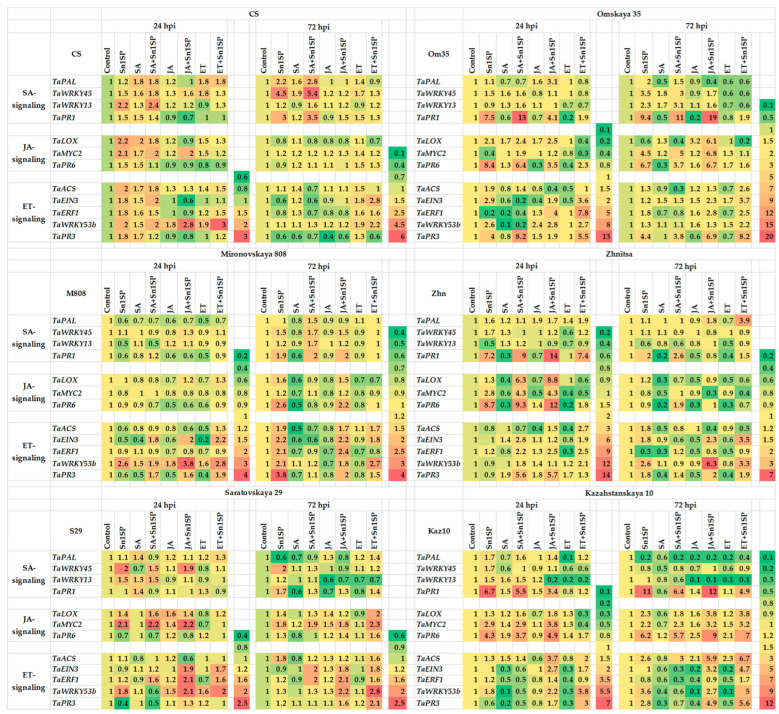
Heat map of relative expression of SA, JA and ethylene signaling pathways genes in six bread wheat cultivars 24 and 72 hpi with the SnTox1-producing isolate *S. nodorum* Sn1SP under the influence of hormonal treatment. Average gene expression was calculated from nine biological samples in five technical replicates (*n* = 9). Values are presented as means.

To study the role of each hormonal signaling pathway (SA, JA or ethylene) in the development of plant resistance or susceptibility to the NE SnTox1, the transcriptional activity of 12 genes of hormonal signaling pathways was studied. The expression of four genes of the SA signaling pathway (*TaPAL*, *TaWRKY13*, *TaWRKY45* and *TaPR1*), three genes of the JA signaling pathway (*TaLOX*, *TaMYC2* and *TaPR6*) and five genes of the ethylene signaling pathway (*TaACS*, *TaEIN3*, *TaERF1*, *TaWRKY53b* and *TaPR3*) were analyzed (Figure 4). We explored the transcript level of genes involved in the biosynthesis of the SA (phenylalanine ammonia-lyase, *TaPAL*), of the JA (lipoxygenase, *TaLOX*) and of the ethylene (aminocyclopropane synthase, *TaACS1*) [11]. We studied the expression of the TF genes. The gene of the SA signaling pathway *TaWRKY13* is an ortholog of the Arabidopsis gene *AtWRKY70* [20] and the *TaWRKY45* gene involved in the NPR1-independent SA signaling pathway. The gene TF of the ethylene signaling pathway *TaEIN3* (Ethylene-Insensitive3) is an ortholog of the Arabidopsis gene *AtEIN3*, and the gene TF of the primary response to the ethylene *TaERF1* (Ethylene Response Factor1) activates the ERF branch of the JA signaling pathway and is responsible for the integration of ethylene signaling pathways and JA [9,11]; the *TaWRKY53b* gene is an ortholog of the Arabidopsis gene *AtWRKY33* [21]; the *TaMYC2* gene is a positive regulator of the expression of JA-dependent genes and controls the MYC branch of the JA signaling pathway [11,22]. According to many studies, *TaWRKY13*, *TaWRKY45*, *TaMYC2*, *TaEIN3*, *TaERF1* and *TaWRKY53b* genes regulate hormone crosstalk and are involved in the plant defense response to pathogens [8,9,11,20,21,22]. Also in this work, the expression of the *TaPR1* gene, which is marker of the SA signaling pathway, the *TaPR3* gene, which is the marker of the ethylene signaling pathway, and the *TaPR6* gene, which is the marker of the JA signaling pathway, were studied [11].

Based on the results of transcriptional analysis, heat maps were compiled for each cultivar (Figure 4).

In susceptible (SnTox1-sensitive) cultivars (CS, Om35), the expression of the JA/ethylene signaling pathway genes and some SA signaling pathway genes was activated 24 hpi, which led to the formation of large zones of damage and the development of susceptibility (Figure 4). The expression of the JA and ethylene biosynthesis genes *TaLOX* and *TaACS*, the TF genes *TaWRKY13*, *TaMYC2*, *TaEIN3*, *TaERF1* and *TaWRKY53b* and the chitinase gene *TaPR3* increased in the cv. CS. Additionally, the transcripts *TaLOX*, *TaACS*, *TaEIN3*, *TaWRKY53b*, *TaPR1*, *TaPR3* and *TaPR6* accumulated in the cv. Om35 24 hpi compared to the control (Figure 4, Figures S1 and S2). The transcript level of the SA biosynthesis gene *TaPAL* and the SA signaling pathway genes *TaWRKY45* and *TaPR1* increased in both cultivars 72 hpi; in addition, the expression of the SA signaling pathway gene *TaWRKY13* and two JA signaling pathway genes *TaMYC2* and *TaPR6* increased in the cv. Om35 (Figure 4, Figures S1 and S2). On the contrary, the expression of some JA and ethylene signaling pathway genes was suppressed in the susceptible cultivars CS and Om35 72 hpi, which led to the formation of large zones of damage and the development of susceptibility (Figure 4). Thus, the expression of the *TaWRKY53b*, *TaLOX* and *TaACS* genes decreased in both cultivars; in addition, in the cv. CS, the transcript level of the *TaPR3* and *TaPR6* genes decreased 72 hpi compared to the control (Figure 4, Figures S1 and S2).

In resistant (SnTox1-insensitive) cultivars (M808, Zhn), the response of the genes was different 24 hpi. Thus, the expression of the SA, JA, and ethylene signaling pathway genes was suppressed in the cv. M808; only the expression of the *TaWRKY53b* gene was activated (Figure 4). At the same time, the expression of the SA and JA signaling pathways genes was activated in the cv. Zhn, as in the susceptible cv. CS (Figure 4). Thus, the transcript level of the *TaPAL*, *TaWRKY45*, *TaPR1*, *TaMYC2* and *TaPR6* genes increased 24 hpi compared to the control (Figure 4, Figures S1 and S2). On the contrary, a predominant increase in the expression of the JA and ethylene signaling pathway genes was found in the resistant cultivars M808 and Zhn 72 hpi, which led to the formation of the smallest lesion zones, comprising only 2–5% of the total leaf area, and led to the development of the resistance (Figure 4). Thus, the transcripts of five genes (*TaACS*, *TaEIN3*, *TaWRKY53b*, *TaPR1* and *TaPR3*) accumulated in both cultivars; in addition, at the same time, the expression of the *TaLOX*, *TaPR6* and *TaERF1* genes increased in the cv. M808 compared to the control (Figure 4, Figures S1 and S2).

In cultivars with nontypical symptoms (S29 and Kaz10), the expression of the SA, JA, and ethylene signaling pathway genes was activated 24 hpi, as in the susceptible cv. CS, only to a lesser extent, which led to the formation of nontypical symptoms (Figure 4). The transcript level of four genes (*TaWRKY13*, *TaWRKY45*, *TaMYC2* and *TaWRKY53b*) increased in both cultivars 24 hpi; in addition, at the same time the expression of the SA biosynthesis gene *TaPAL* and two genes of the PR proteins *TaPR1* and *TaPR6* increased in the cv. Kaz10 compared to the control (Figure 4, Figures S1 and S2). Interestingly, cultivars S29 and Kaz10 responded differently 72 hpi. The expression of the SA and JA signaling pathway genes was weakly activated in cv. S29, and the ethylene signaling pathway was suppressed, while the expression of four genes (*TaWRKY45*, *TaPR1*, *TaMYC2* and *TaACS*) was activated compared to the control (Figure 4, Figures S1 and S2). During this period, the expression of the JA and ethylene signaling pathway genes was activated in the cv. Kaz10 (Figure 4), as in the resistant cultivars M808 and Zhn (Figure 4). The expression of seven genes (*TaLOX*, *TaACS*, *TaMYC2*, *TaWRKY53b*, *TaPR1*, *TaPR6* and *TaPR3*) increased in the cv. Kaz10 72 hpi compared to the control (Figure 4, Figures S1 and S2). In cultivars S29 and Kaz10, an unusual resistant genotype pattern of the gene expression of hormonal signaling pathways was observed 24 and 72 hpi, which was most likely associated with the development of nontypical symptoms.

In general, these results indicate that the expression pattern of genes encoding the biosynthetic enzymes of phytohormones *TaPAL*, *TaLOX* and *TaACS* and the TF genes of hormonal signaling pathways, as well as the *TaPR3* gene encoding chitinase, depended on the wheat genotype and on the sensitivity of the cultivar to the NE SnTox1 and was associated with the development of the susceptibility or resistance of cultivars to the disease. The expression of two PR genes (*TaPR1* and *TaPR6*) depended on the genotype of the cultivar, but it was not associated with the sensitivity of the cultivar to the NE SnTox1.

Further in the work, we studied the effect of treatment with hormones on the induction or suppression of signaling pathways in plants infected with the SnTox1-producing isolate of S*. nodorum* Sn1SP (Figure 4).

Treatment with SA activated the expression of the SA and JA signaling pathway genes in susceptible cultivars (CS and Om35) and resistant cultivars with nontypical symptoms (S29 and Kaz10) 24 hpi (Figure 4). In addition, at the same time, treatment with SA inhibited the expression of ethylene signaling pathway genes in the susceptible cv. Om35 and cultivars with nontypical symptoms (S29 and Kaz10) compared to the control, which could lead to an increase in the damage area by 7–14% and the development of susceptibility (Figure 4, Table 1). On the contrary, during this period, treatment with SA did not induce the expression of the SA signaling pathway genes in the resistant cultivars M808 and Zhn but increased the transcript level of the ethylene signaling pathway genes in both cultivars and JA signaling pathway genes in the cv. Zhn compared to the control and untreated SA and infected Sn1SP plants (Figure 4). Treatment with SA activated the expression of SA signaling pathway genes in susceptible cultivars (CS and Om35), did not affect the transcript level of SA-dependent genes in resistant cultivars M808 and Zhn, and suppressed the accumulation of transcripts of SA signaling pathway genes in resistant cultivars with nontypical symptoms (S29 and Kaz10) 72 hpi compared to plants untreated with SA and infected with Sn1SP (Figure 4). The treatment with SA did not affect the expression of JA-dependent genes in susceptible cultivars (CS and Om35) and in resistant cultivars with nontypical symptoms (S29 and Kaz10) but suppressed the transcript accumulation of JA signaling pathway genes in resistant cultivars M808 and Zhn 72 hpi compared to infected plants untreated with SA (Figure 4). The SA treatment suppressed the ethylene signaling pathway in all cultivars except the cv. S29 72 hpi compared to the control and infected plants untreated with SA (Figure 4). Such reprogramming of gene expression led to the development of susceptibility in all six wheat genotypes (Figure 1).

The treatment of plants with JA induced the expression of JA and ethylene signaling pathway genes in five cultivars (CS, Om35, Zhn, S29 and Kaz10) 24 hpi, which could lead to a reduction in the damage area and the development of resistance (Figure 4). JA treatment induced only the ethylene signaling pathway genes in the cv. M808 24 hpi, which did not affect the size of the affected areas (Figure 4). In addition, JA treatment inhibited the expression of SA-dependent genes in susceptible cultivars 24 and 72 hpi, and we observed a reduction in disease symptoms by 30–42% (Figure 4, Table 1). JA treatment inhibited the expression of SA signaling pathway genes in the resistant cultivar with nontypical symptoms, i.e., Kaz10, and did not affect the expression of SA-dependent genes in the other three resistant cultivars M808, Zhn and S29 72 hpi compared to the infected plants untreated with JA (Figure 4). However, we observed a decrease in disease symptoms by only 3–6% in resistant cultivars with nontypical symptoms (S29 and Kaz10) (Table 1). At the same time, treatment with JA induced the expression of the JA signaling pathway genes in two cultivars Om35 and Kaz10 and of the ethylene signaling pathway genes in cultivars S29 and Kaz10 compared to the infected plants untreated with JA, which, most likely, could lead to a reduction in the damage area (Figure 4). Under the same conditions, the expression of only one gene of the ethylene signaling pathway, *TaWRKY53b*, was induced in the cv. Zhn, while the expression of the *TaPR3* gene was activated in the cv. Om35 (Figure 4).

The treatment of plants with ethephon (ET) induced the expression of the ethylene signaling pathway genes and inhibited the expression of the SA and JA signaling pathway genes 24 hpi in all cultivars compared to the control and infected plants untreated with ET (Figure 4). Treatment with ET also induced the ethylene signaling pathway in all cultivars 72 hpi (Figure 4). In addition, treatment with ET suppressed the expression of SA signaling pathway genes in susceptible (SnTox1-sensitive) cultivars (CS and Om35) 72 hpi compared to infected plants untreated with ET, which could lead to a reduction in the affected areas and an increase in resistance (Figure 4). The treatment with ET did not affect the expression of the SA signaling pathway genes but suppressed the expression of the JA signaling pathway genes in resistant (SnTox1-insensitive) cultivars (M808 and Zhn) 72 hpi compared to the control, which could lead to an increase in lesions on the leaves of these cultivars (Figure 4). The ET treatment suppressed the SA signaling pathway but activated the JA signaling pathway in resistant cultivars with nontypical symptoms (S29 and Kaz10) 72 hpi compared to the control, which could affect the resistance of these cultivars (Figure 4).

The effect of treatment with SA, JA and ET on the expression of the hormone biosynthesis genes *TaPAL*, *TaLOX* and *TaACS* and the expression of *PR* genes (*TaPR1*, *TaPR3* and *TaPR6*) depended on the genotype of the cultivar, but it was not associated with the sensitivity of the cultivars to the NE SnTox1 (Figure 4). Thus, SA treatment did not affect the expression of the studied six genes in three cultivars CS, S29 and Kaz10 but decreased the expression of *TaLOX* and *TaACS* and increased the expression of *TaPR1* and *TaPR3* in three other cultivars Om35, M808 and Zhn; additionally, it decreased the expression of *TaPR6* only in two cultivars Om35 and M808 compared to the control (Figure 4). Treatment with JA or ET did not effect, or had a weak effect, on the expression of hormone biosynthesis genes and *PR* genes in two cultivars M808 and S29, except for the expression of the *TaPR3* gene, and had a negative effect on the induction of the expression of these genes in the cv. CS (Figure 4). The expression pattern of the *TaPAL* and *TaACS* genes in the three cultivars Om35, Zhn and Kaz10 treated with JA or ET and the expression pattern of the *TaPR1* and *TaPR6* genes in the three cultivars Om35, Zhn and Kaz10 treated with JA had varietal characteristics and did not depend on sensitivity to the NE SnTox1 (Figure 4). JA treatment induced the expression of the *TaLOX* gene in three cultivars Om35, Zhn and Kaz10 (Figure 4). Treatment with ET or JA induced the expression of the *TaPR3* gene in five cultivars Om35, M808, Zhn, S29 and Kaz10 (Figure 4). Treatment with ET inhibited the expression of the *TaLOX*, *TaPR1* and *TaPR6* genes in all six cultivars (Figure 4).

#### 2.3.2. Analysis of the Expression of the Gene Encoding of Hormonal Signaling Pathway Transcription Factors in Various Wheat Genotypes

**Figure 5:**
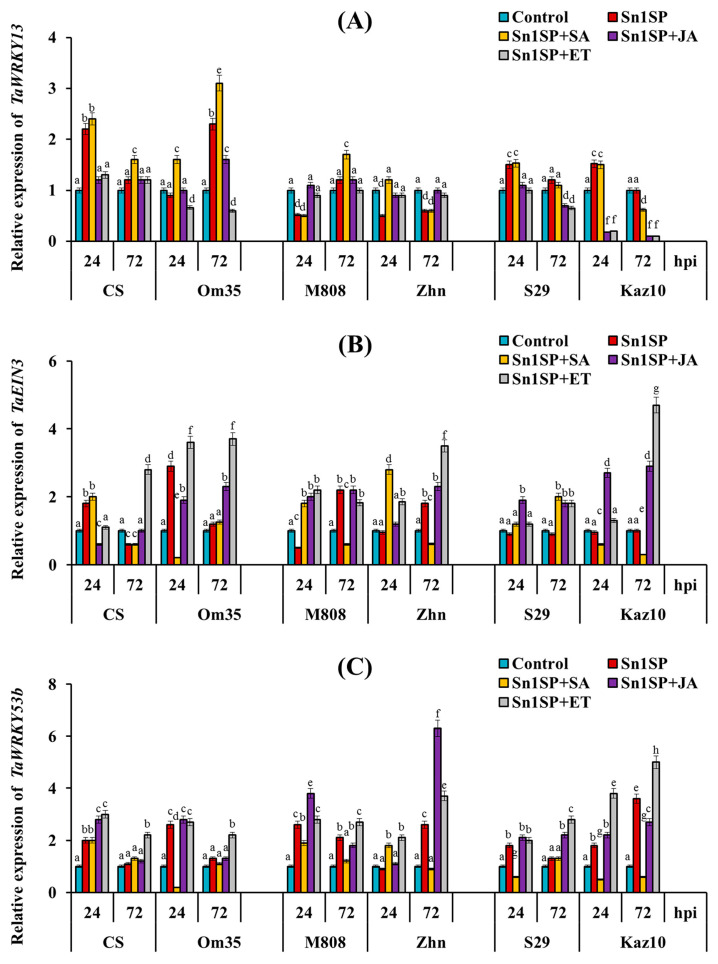
Effect of SA, JA and ET treatment on the expression of three TF genes—*TaWRKY13* (**A**), *TaEIN3* (**B**) and *TaWRKY53b* (**C**) in six bread wheat cultivars 24 and 72 hpi with the SnTox1-producing isolate *S. nodorum* Sn1SP. Symbols are the same as in Figure 3. Average gene expression was calculated from nine biological samples in five technical replicates (*n* = 9). Values are expressed as mean SE (ANOVA with Duncan’s test; comparisons between genotypes are presented; significant differences are marked with different letters at *p* ≤ 0.05).

The effect of treatment with SA, JA and ET on the expression of the TF genes of hormonal signaling pathways depended on the sensitivity of cultivars to the NE SnTox1 (Figure 4). Let us consider in more detail the expression of three TFs genes—*TaWRKY13*, *TaEIN3* and *TaWRKY53b* (Figure 5).

The treatment of plants with SA increased the expression of the *TaWRKY13* gene 24 and 72 hpi in susceptible (SnTox1-sensitive) cultivars (CS and Om35) and did not affect the expression of the *TaWRKY13* gene in resistant cultivars with nontypical symptoms (S29 and Kaz10) but increased the level of the transcripts of this gene in the resistant cultivar M808 72 hpi compared to the control (Figure 5). The treatment of plants with SA increased the expression of the *TaEIN3* gene 24 hpi and decreased it 72 hpi in resistant (SnTox1-insensitive) cultivars (M808 and Zhn) compared to the control, which could lead to an increase in the damage area by 7.5 and 29.4%, respectively (Figure 5, Table 1). Also, the treatment of plants with SA reduced the expression of the *TaWRKY53b* gene 24 and 72 hpi in four resistant cultivars M808, Zhn, S29 and Kaz10 compared to the control, which could lead to an increase in the damage area by 7.5, 29.4, 13.6 and 7.3%, respectively (Figure 5, Table 1).

The treatment of plants with JA reduced the expression of the *TaWRKY13* gene 24 and 72 hpi in susceptible (SnTox1-sensitive) cultivars (CS and Om35) and in resistant cultivars with nontypical symptoms (S29 and Kaz10), which could lead to a reduction in the damage area by 42.2, 30.4, 3.4 and 6.3%, respectively (Figure 5, Table 1). The expression of the *TaWRKY13* gene in the resistant (SnTox1-insensitive) cultivars M808 and Zhn treated with JA remained at the control level 24 and 72 hpi (Figure 5). The JA treatment increased the expression of the *TaWRKY53b* gene in three cultivars CS, Om35 and M808 24 hpi, as well as the mRNA content of the *TaWRKY53b* gene enhanced in the JA-treated cv. Zhn after 72 h of infection and in cultivars with nontypical symptoms, i.e., S29 and Kaz10 24, 72 hpi compared to the control (Figure 5). Treatment with JA increased the expression of the *TaEIN3* gene in the susceptible cv. Om35 and in cultivars with nontypical symptoms, i.e., S29 and Kaz10, which could lead to a reduction in the damage area (Figure 5).

The treatment of plants with ET affected the expression of the *TaWRKY13* gene in the same way as the treatment of plants with JA (Figure 5). The treatment of plants with ET increased the expression of the *TaEIN3* and *TaWRKY53b* genes in all cultivars 24 and 72 h after infection compared to the control (Figure 5).

#### 2.3.3. Analysis of the Expression of Genes of MAPK in Various Wheat Genotypes

**Figure 6:**
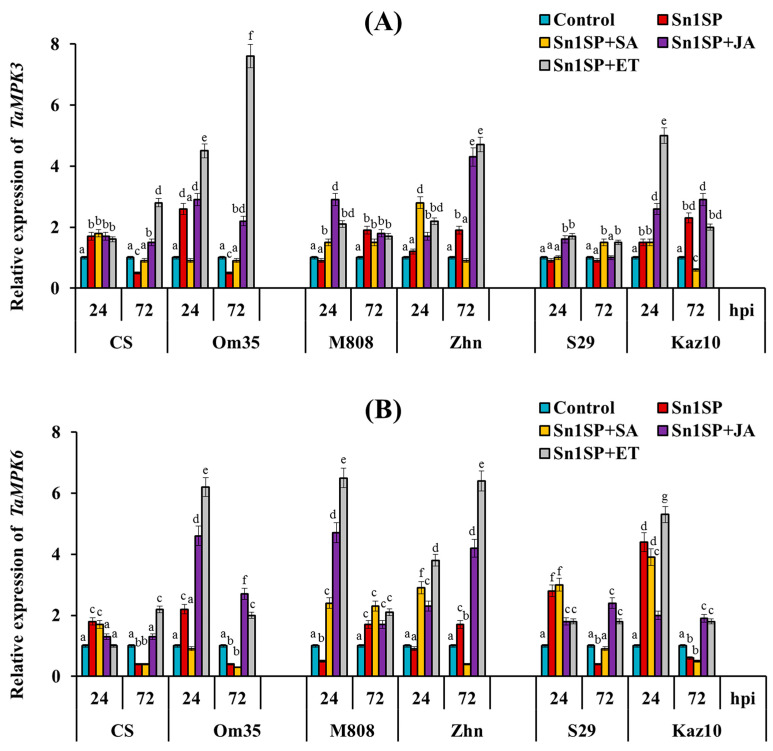
Effect of SA, JA and ET treatment on the expression of two genes *TaMPK3* (**A**) and *TaMPK6* (**B**) in six bread wheat cultivars 24 and 72 hpi with the SnTox1-producing isolate *S. nodorum* Sn1SP. Symbols are the same as in Figure 3. Average gene expression was calculated from nine biological samples in five technical replicates (*n* = 9). Values are expressed as mean SE (ANOVA with Duncan’s test; comparisons between genotypes are presented; significant differences are marked with different letters at *p* ≤ 0.05).

It was previously shown that SnTox1 activated the MAPK cascade [16], so we studied the expression of two genes *TaMPK3* and *TaMPK6* in six bread wheat cultivars treated with hormones and infected with the SnTox1-producing isolate *S. nodorum* Sn1SP (Figure 6). The expression of the *TaMPK3/TaMPK6* module was upregulated in susceptible (SnTox1-sensitive) cultivars (CS, Om35) 24 hpi and decreased 72 hpi compared to the controls (Figure 6). On the contrary, in the resistant (SnTox1-insensitive) cultivars (M808 and Zhn), the expression of the *TaMPK3/TaMPK6* module was not activated or even inhibited 24 hpi, and it was activated 72 hpi (Figure 6). In cultivars with nontypical symptoms (S29 and Kaz10), different expression patterns of the *TaMPK3* and *TaMPK6* genes were found (Figure 6). Thus, in the cv. S29, the expression of the *TaMPK3* genes did not change 24 and 72 hpi, and the expression of the *TaMPK6* gene increased 24 hpi and decreased 72 hpi, as in susceptible cultivars (Figure 6). In the cv. Kaz10, the expression of the *TaMPK3/TaMPK6* module was activated 24 hpi compared to the control, as in susceptible cultivars (Figure 6). However, in this cultivar, the expression of the *TaMPK6* gene decreased, as in susceptible cultivars, and the expression of the *TaMPK3* gene increased, as in resistant cultivars, 72 hpi compared to the control (Figure 6).

The treatment of plants with SA increased the expression of the *TaMPK3/TaMPK6* module in four cultivars CS, M808, Zhn and Kaz10 24 hpi compared to the control, as in susceptible cultivars (Figure 6). The expression of the *TaMPK3/TaMPK6* module was not activated or decreased in the SA-treated plants of five cultivars (CS, Om35, Zhn, S29 and Kaz10) 72 hpi compared to the control as in susceptible cultivars (Figure 6). The treatment of plants with JA or ET similarly affected the expression of the *TaMPK3/TaMPK6* module in six cultivars (Figure 6).

The expression of the *TaMPK3/TaMPK6* module increased in the JA-treated plants of four cultivars (Om35, M808, Zhn and Kaz10) 24 and 72 hpi compared to the control (Figure 6). In the cv. S29, JA treatment activated the *TaMPK3/TaMPK6* module only 72 hpi compared to the control (Figure 6). JA treatment increased *TaMPK3* gene expression and did not increase *TaMPK6* gene expression in cv. CS (Figure 6). The expression of the *TaMPK3/TaMPK6* module increased in ET-treated plants of five cultivars (Om35, M808, Zhn, S29 and Kaz10) 24 hpi and in all six cultivars 72 hpi compared to the control (Figure 6).

#### 2.3.4. Correlation Analysis of Key Parameters in Various Wheat Genotypes

**Figure 7:**
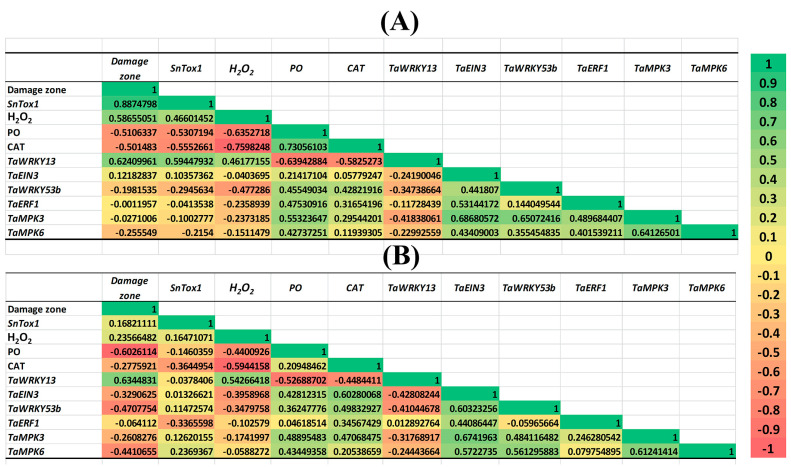
Correlation matrix representing the correlation between key parameters examined in this study in six cultivars treated with SA, JA and ET: expression of the *SnTox1* gene, MAPK genes and TF genes of hormonal signaling pathways, damage zone, H_2_O_2_ content and activity of PO and CAT after 24 (**A**) and 72 (**B**) hours of infection.

All parameters studied in this work in six cultivars of wheat treated with SA, JA and ET were analyzed using correlation analysis, which showed that the H_2_O_2_ content, the expression of the NE *SnTox1* gene and the TF gene *TaWRKY13* 24 hpi were positively correlated with the size of the damage areas, and the antioxidant activity of the cultivars (the activity of PO and CAT enzymes) negatively correlated with these indicators (Figure 7).

Interestingly, the expression of the TF genes *TaWRKY53b*, *TaEIN3* and *TaERF1* and the *TaMPK3* and *TaMPK6* genes 24 and 72 hpi positively correlated with the activity of the antioxidant enzymes PO and CAT and negatively correlated with the H_2_O_2_ content, the expression of the *TaWRKY13* gene and the size of the damage areas (Figure 7).

## 3. Discussion

### 3.1. The Influence of Phytohormones on the SnTox1-Snn1 Interaction in Different Wheat Genotypes

Plant pathogens secrete a large arsenal of effectors that interfere with plant defenses and promote pathogen colonization [1,5,6]. Given the importance of phytohormonal pathways in plant immunity, it is not surprising that plant pathogens release effectors that target hormonal pathways [5,6]. The influence of effectors on hormonal signaling pathways has been well studied in the biotrophic fungus *Ustilago maydis*, necrotrophic fungi *Sclerotinia sclerotiorum*, *Cochliobolus victoria* and *B. cinerea*, hemibiotrophic rice blast fungus *Magnaporthe oryzae*, oomycete pathogens *Phytophthora* spp., and some other pathogens [5,6]. However, the effectors of *S. nodorum* (synonym *Parastagonospora nodorum*) in this regard have been little studied. It was previously shown that the *S. nodorum* effectors ToxA and Tox3 influence the SA signaling pathway through directly interacting with the PR1 protein, which can lead to increased susceptibility to *S. nodorum* [23]. We previously showed that SnTox3 activated the ethylene signaling pathway to reduce the cytokinin content in plants, which led to the development of susceptibility [13]. The effect of the SnTox1 effector on hormonal signaling pathways has not been studied.

The NE SnTox1 is widely distributed among *S. nodorum* isolates worldwide [24], and the SnTox1-*Snn1* interaction explains up to 58% of disease variation [14]. In this work, treatment with the SA, JA and ET (a chemical precursor of ethylene) of six cultivars of bread wheat with varying sensitivity to NE SnTox1 was used to study the role of SnTox1 in manipulating the SA, JA and ethylene signaling pathways and to reveal the role of the hormone’s crosstalk in the development of the resistance/susceptibility of wheat to the SnTox1-producing isolate *S. nodorum*.

In this work, we used the SnTox1-sensitive wheat cultivar Chinese Spring (CS), in which the susceptibility gene *Snn1*, encoding the transmembrane WAK (wall-associated kinase) receptor, had previously been cloned and characterized [17]. Using transgenesis and mutagenesis, it has been proven that mutations in the *Snn1* gene lead to the loss of sensitivity to the NE SnTox1 and make the cultivar resistant to the disease caused by a SnTox1-producing isolate [17]. Previously, it has been discovered that the cv. M808 is insensitive to NE SnTox1 [17]. The wheat cultivars Om35, Kaz10, S29 and Zhn were selected based on our previous results [18]. It is worth noting that all six cultivars carried the *Snn1* gene allele [18]. Two cultivars CS and Om35 showed sensitivity to NE SnTox1; extensive damage areas were found in them (Table 1). The four cultivars M808, Zhn, S29 and Kaz10 showed insensitivity to NE SnTox1 (Figure 1). Nontypical symptoms of damage were found in two cultivars Kaz10 and S29 (Figure 1); these symptoms were similar to “flecking” [19]. It is interesting to note that Xu et al. (2004) found a significant correlation between SNB resistance and “flecking” caused by the SnTox1-*Snn1* interaction in synthetic hexaploid wheat lines [25].

The diversity of the manifestations of one SnTox1-*Snn1* interaction may be caused by different levels of NE gene expression during the infection of a plant sample [26]. The results of this work showed that the expression of the *SnTox1* gene in the Sn1SP isolate depended on the degree of the sensitivity of the cultivar to NE and was higher during the infection of susceptible cultivars CS and Om35 than during the infection of resistant cultivars (Figure 2), which coincides with the results of other researchers [19]. In addition, correlation analysis showed a positive dependence of the *SnTox1* gene expression level on the size of the affected areas and proved the regulatory role of the phytohormones SA, JA and ethylene in this process (Figure 2 and Figure 7). Treatment with SA generally increased the expression of the *SnTox1* gene, which could lead to an increase in the damage area, whereas treatment with JA or ET decreased or had no effect on the expression of *SnTox1* gene, which could lead to a reduction in the damage area (Figure 2). We hypothesize that one of the mechanisms for regulating the transcription of NE genes may be associated with the activity of hormonal signaling pathways that can indirectly positively or negatively regulate the expression of NE genes according to the feedback principle.

### 3.2. SnTox1 Manipulates Hormonal Signaling Pathways to Regulate Plant Redox Status and Necrosis Formation

The formation of necrosis on wheat leaves under the action of the SnTox1-producing isolates of *S. nodorum* is closely related to the effect of NE on the production of the ROS and redox metabolism of plants [16]. It should be noted that there are strong differences regarding the role of ROS and antioxidants in the development of plant resistance to necrotrophic and biotrophic pathogens [27]. A sharp and multiple increase in ROS content, leading to an oxidative burst and the death of plant cells, stops the growth of biotrophic pathogens but promotes the colonization of plant tissues by necrotrophs [27,28]. The results of this work showed that a wave in the formation of ROS in susceptible cultivars CS and Om35 24 hpi, leading to the formation of necrosis, occurred due to a decrease in CAT activity and the absence of an increase in PO activity, which is proven by the opposite reaction of resistant cultivars (Figure 3). In addition, it is known that peroxidases generate ROS together with NADPH oxidases but can also utilize ROS to form lignin polymers in the apoplast during plant–microbial interaction, and catalases degrade H_2_O_2_ [29]. Also, the effect of fungal toxins on plant CATs was shown using the example of a mycotoxin from fungi of the genus *Fusarium* fusaric acid (FA), which induced the generation of ROS in tomato plants for the formation of necrosis by reducing CAT activity [28].

However, it is known that phytohormones regulate the formation of ROS [27]. We hypothesize that NE SnTox1 can manipulate the hormonal signaling pathways of SA, JA and ethylene in wheat plants to regulate plant redox status and necrosis formation. A study of the redox status in six different wheat genotypes treated with SA, JA or ET showed that the effect of plant treatment with SA was aimed at reducing CAT activity to increase the H_2_O_2_ content and the development of large lesion zones (Figure 3), which is consistent with the results of other authors [30]. It is known that SA and ROS are two important signaling components that activate PCD [31]. In addition, SA is an inhibitor of the activity of catalase, the main enzyme that catalyzes the destruction of H_2_O_2_, which leads to an increase in the concentration of H_2_O_2_, the development of an oxidative burst, the formation of necrosis and the proliferation of necrotrophic pathogens [30,32,33]. Thus, the work of other authors showed the effect of increased SA content on the inhibition of CAT2 activity, which led to increased susceptibility of *Arabidopsis* plants to the necrotroph *B. cinerea* [30].

The effect of JA and ET in this study was aimed at reducing the H_2_O_2_ content by increasing the activity of PO and CAT, which led to a decrease in the affected areas (Figure 3). The only exception was the cv. Zhn, in which, under the influence of ET treatment, CAT activity greatly decreased, but PO activity increased (Figure 3). Our results are consistent with the results of other authors about an increase in the antioxidant capacity of plants under the influence of JA or ethylene [22]. Thus, the treatment of plants with JA significantly reduced the H_2_O_2_ content and lipid peroxidation in wheat infected with *Fusarium culmorum* and led to increased resistance [34]. Many studies have shown that JA positively regulates the expression of certain peroxidase genes [35,36,37]. JA treatment caused a significant activation of PO, which led to the enhanced protection of wheat plants against *Fusarium graminearum* [38]. Another study showed that JA biosynthesis is closely related to CAT2 activity and the resistance of *Arabidopsis* plants to *B. cinerea* [39]. CAT2-N overexpressing *Arabidopsis* plants have increased JA accumulation and enhanced resistance to *B. cinerea* [39].

Unfortunately, the mechanisms of ethylene’s influence on the processes of ROS generation under biotic stress have not been sufficiently studied [27,28,40,41]. Although in some cases infection was accompanied by a parallel accumulation of ethylene and H_2_O_2_ [42], it remains unclear whether ethylene induced the synthesis of H_2_O_2_. The role of ethylene in the regulation of catalase activity is not fully understood, especially under biotic stress, but some studies have shown that ethylene is capable of activating CAT [41,42,43,44]. Thus, the simultaneous accumulation of ethylene and an increase in CAT activity were observed in forms of barley susceptible to powdery mildew [27]. In transgenic plants with an impaired synthesis or reception of ethylene, a decrease in CAT activity was found [27,28,41]. We have previously shown that increased CAT activity is a characteristic feature of wheat plants treated with ET and infected with *S. nodorum* and is the cause of low H_2_O_2_ production in wheat at the early stages of infection [45]. The influence of ethylene on the activity of PO during plant infection has also been little studied; most studies of the interaction of ethylene and PO are devoted to the processes of growth and development, fruit ripening, or the action of abiotic stress factors [43,44]. Many studies have shown that ethylene activated the PO [46,47]. Recent work on wild-type (WT) and ethylene receptor mutant *Never ripe* (*Nr*) tomato plants has proven the role of ethylene in the induction of plant antioxidant enzymes PO, CAT and superoxide dismutase for ROS detoxification under the influence of the mycotoxin fusaric acid (FA) [28].

Thus, our results indicate that NE SnTox1 could influence the redox enzymes PO and CAT through the antagonism of the SA and JA/ethylene hormonal signaling pathways. Thus, recent studies have shown that CATALASE2 (CAT2) promotes JA-biosynthetic acyl-CoA oxidase (ACX) activity, thereby enhancing JA biosynthesis, and SA inhibits CAT2 to suppress the JA biosynthesis and signaling pathway [30,39]. NE SnTox1 could hijack the SA signaling pathway and use it to suppress CAT activity, accumulate H_2_O_2_, and form necrosis and the simultaneous suppression of the JA and ethylene hormonal pathways using SA.

### 3.3. SnTox1 Uses SA and JA/Ethylene Antagonism to Reprogram TF Gene Expression

Recently, the role of hormones in plant immunity is considered within the framework of the concept of hormonal networks or hormone crosstalk, where it is believed that antagonistic or synergistic interactions between hormonal pathways allow the plant to exactly regulate its immune response depending on the lifestyle of the pathogen [3]. However, recent research also shows that the defense response in the hormonal network produces multiple signals and involves multiple genes, leading to pleiotropic effects or the “ambivalence effect.” In the ambivalence effect, gene–hormone relationships lead to both positive and negative effects of immune responses due to the diversity of plant pathogen lifestyles [3,46,48,49,50,51,52,53]. An example of the ambivalence effect is the antagonism between the SA and JA/ethylene pathways, which has been demonstrated in many plant species [11]. In recent years, it has been shown that there are pathogen effectors that directly influence the antagonism of the SA and JA/ethylene hormonal pathways, using such tactics to combat host immune responses [3,31].

The results of this work showed that the activation or suppression of the gene expression of hormonal signaling pathways depended on the sensitivity of the cultivar to NE SnTox1 (Figure 4). The activation of SA signaling and the suppression of JA/ethylene signaling, which was found in SnTox1-sensitive cultivars, were associated with H_2_O_2_ accumulation, necrosis formation and susceptibility development, as shown by the results and correlation analysis (Figure 7). The activation of JA/ethylene signaling pathways, which was found in SnTox1-insensitive cultivars, was associated with an increase in plant antioxidant activity, a decrease in H_2_O_2_ content and the development of resistance (Figure 7).

Taken together, our data of the gene expression and redox status of resistant and sensitive genotypes treated with SA, JA and ET showed that SnTox1 exploited antagonistic interactions between SA and JA/ethylene signaling pathways for pathogen development and propagation (Figure 3, Figure 4 and Figure 7). The treatment of plants with SA affected gene expression depending on the sensitivity of the cultivar to SnTox1. Thus, SA activated the expression of SA pathway genes and suppressed the expression of ethylene pathway genes in SnTox1-sensitive cultivars and suppressed the expression of JA pathway genes in SnTox1-insensitive cultivars (Figure 4). The results of treating plants with SA showed that it was the induction of the expression of SA pathway genes and the suppression of the expression of JA pathway genes 72 hpi that were important for the development of susceptibility to the disease (Figure 4). The treatment of plants with SA or its synthetic analogues (2,6-dichloroisonicotinic acid (INA), benzothiadiazole (BTH), probenazole, PBZ and others) is actively used against viruses and biotrophic and hemibiotrophic pathogens [54]. PBZ was applied to *Oryza sativa* to prevent rice blast caused by *Magnaporthea grisea*; INA was applied to *Cucumis sativus* and *Nicotiana tabacum* to prevent anthracnose (caused by *Colletotrichum lagenarium*) and tobacco mosaic virus infection, respectively [54,55]. However, there is very little data on the role of SA signaling in plant response to necrotrophic pathogens. Breen et al. (2016) showed that the *S. nodorum* effectors ToxA and Tox3, by manipulating the SA signaling pathway, caused the increased susceptibility of plants to the *S. nodorum* [23]. The study of such wheat pathogens as *Pyrenophora tritici-repentis* and *S. nodorum* may provide novel opportunities for examining the role of SA signaling in plant response to necrotrophic pathogens. We assume that the pathogen *S. nodorum*, using SnTox1, could activate the SA signaling pathway to suppress the induction of the ethylene and JA signaling pathways directed against necrotrophic pathogens, which is confirmed by the results of other authors [8,9]. It was proved that the main regulatory component of the SA signaling pathway, the protein NPR1 (from the nonexpresser of PR genes1), physically interacted with TF EIN3 and, thereby, inhibited the expression of ethylene-dependent genes [56]. The necrotrophic pathogen *B. cinerea* produces an exopolysaccharide acting as an activator of the SA signaling pathway in tomato plants and suppressing the JA signaling pathway and the expression of protease inhibitor genes (*PR6*) necessary for the resistance of tomato plants to *B. cinerea* [57]. Also, the effector protein of the necrotrophic fungal pathogen *Sclerotinia sclerotiorum* SSITL (sclerotinia sclerotiorum integrin-like) suppresses JA-dependent defense, increasing plant susceptibility [58].

The treatment of plants with JA led to the suppression of the expression of SA signaling pathway genes in SnTox1-sensitive cultivars 24 and 72 hpi, which was associated with the development of resistance (Figure 4). However, the results of treating other wheat genotypes with JA showed that it suppressed the expression of SA pathway genes 72 hpi, which was important for the development of plant resistance to the disease (Figure 4). Additional studies on another plant species have shown that the activation of the JA pathway suppresses SA biosynthesis and the SA-dependent response [9]. In addition, many studies have shown that the JA pathway is involved in the development of resistance to such necrotrophic pathogens as *A. brassicicola*, *D. dadanii*, *B. cinerea* and others [22,57,59]. The treatment of plants with JA or MeJA led to the enhanced protection of wheat plants against *F. culmorum* and *F. graminearum* [34,38]. The treatment of *Pinus sylvestris* seedlings with MeJA led to increased resistance to both fungal pathogens *Heterobasidion annosum* and *Lophodermium seditiosum* and insect pests *Neodiprion sertifer* and *Hylobius abietis* [60].

The effect of ethylene on the expression of the genes of hormonal signaling pathways depended on the sensitivity of the cultivar to SnTox1. Thus, the treatment of plants with ET led to the suppression of the expression of the SA signaling pathway genes in SnTox1-sensitive cultivars and cultivars with nontypical symptoms after 72 h of infection, which was associated with the development of resistance (Figure 4). At the same time, the ethylene signaling pathway was activated in all cultivars (Figure 4). The antagonism of ethylene and SA is confirmed by studies on the ethylene-insensitive double mutants of *A. thaliana ein3-1/eil1-1*, where mutants could accumulate a significant amount of SA and demonstrated increased resistance to the hemibiotrophic bacterium *P. syringae* [61]. We hypothesize that the activation of the ethylene signaling pathway was most likely associated with the development of resistance to the SnTox1-producing *S. nodorum* isolate. Our hypothesis is confirmed by the results of other authors. It has been shown that the ethylene signaling pathway is involved in the defense response of *A. thaliana* against *B. cinerea* through its effects on calcium-dependent protein kinases (CPK5, CPK6 and CPK11) [62]. Ethylene-insensitive soybean mutants showed increased susceptibility to the necrotrophic fungus *Rhizoctonia solani* and *A. thaliana* mutants with reduced ethylene sensitivity developed susceptibility to the necrotrophic fungus *B. cinerea* [63,64]. In addition, ET treatment activated the expression of JA pathway genes in cultivars with nontypical symptoms after 72 h of infection, which was associated with the development of resistance (Figure 4). However, the treatment of plants with ET led to the suppression of the expression of JA signaling pathway genes in SnTox1-insensitive cultivars after 72 h of infection, which was associated with the development of susceptibility (Figure 4). Recent work has shown that the interaction between two TFs of the ethylene and JA signaling pathways EIN3 and MYC2, respectively, can be both synergistic and antagonistic, with EIN3 playing a dominant role in this interaction [65], which may determine antagonistic or synergistic interactions between these two pathways. Thus, the results of treating plants with ET showed that it was the activation of the expression of JA pathway genes 72 hpi that was important for the development of plant resistance to the disease (Figure 4).

Thus, our results proved that the SA signaling pathway is involved in the development of susceptibility, the JA signaling pathway is involved in the development of the resistance of wheat plants to the SnTox1-producing isolate of *S. nodorum* and the effect of ethylene may be different and depend on the presence or absence of a compatible interaction gene-for-gene SnTox1-*Snn1*. At the same time, NE SnTox1 uses antagonistic interactions between SA and JA/ethylene signaling pathways to manipulate these pathways.

To further decode the role of phytohormones in the development of the resistance/susceptibility of wheat plants to the pathogen *S. nodorum* and how the SnTox1 manipulates hormonal signaling pathways, we analyzed the expression of TF and MAPK genes and carried out a correlation analysis (Figure 5, Figure 6 and Figure 7). Recent studies have shown that the interaction of hormonal signaling pathways occurs at the transcriptional level through target genes, which are most often the genes of the TFs of such large families as WRKY, ERF, MYC, MYB and NAC [3]. The activation of the MAPK cascade has been shown in many plants during pathogen attack and in hormonal signaling [66,67]. In this case, TFs can serve as substrates for MAPKs [68]. Previous studies have shown that the TF genes *MYC2*, *ERF1*, *WRKY70*, *WRKY33* and others are involved in the antagonism of the SA-JA pathways, the genes *EIN3*, *ERF1* and *MYC2* are involved in the interaction of the JA and ethylene pathways, and *EIN3* is involved in the antagonism of SA and ethylene [3,20,22,25,32,61,65]. Our results showed that *TaWRKY13* (an ortholog of *AtWRKY70*) activated the SA pathway and could inhibit the ethylene/JA signaling pathway in SnTox1-sensitive cultivars. On the contrary, *TaWRKY53b* (an ortholog of *AtWRKY33*) could suppress the SA signaling pathway and activate the ethylene signaling pathway in SnTox1-insensitive cultivars, which was confirmed by hormonal treatments (Figure 5 and Figure 7). *TaEIN3* could inhibit the SA signaling pathway and, together with *TaERF1*, activate the ERF branch of the JA pathway in SnTox1-insensitive cultivars (Figure 5 and Figure 7). Correlation analysis showed a negative relationship between the expression of two ethylene signaling pathway genes *TaWRKY53b* and *TaEIN3* and the SA pathway gene *TaWRKY13* in all six cultivars under all hormonal treatments, and this negative correlation was more significant 72 hpi (Figure 7). In addition, *TaMPK3* and *TaMPK6* most likely induced the ethylene signaling pathway through the activation of *TaWRKY53b* expression, which led to the suppression of oxidative burst. These results are confirmed by the correlation analysis carried out in this study (Figure 7) and data from other studies in model plants [68]. Taken together, our results prove that NE SnTox1 manipulated the antagonism of the SA and JA/ethylene signaling pathways by reprogramming the expression of the MAPK genes *TaMRK3* and *TaMRK6* and the TF genes *TaWRKY13*, *TaEIN3* and *TaWRKY53b*.

Our study provides additional results into the regulation of hormonal crosstalk between SA and JA/ethylene signaling pathways in the development of wheat resistance to *S. nodorum* and other necrotrophic pathogens. Antagonistic effects between SA and JA/ethylene signaling pathways are thought to enable plant survival against pathogens with different lifestyles by shifting defense responses to either the SA or JA/ethylene signaling pathways according to the type of stress, which is called the ambivalence effect [3]. However, pathogens have evolved the ability to manipulate the ambivalence effect by secreting different effectors. The effectors of *M. oryzae*, MoHTR1 and MoHTR2, which modulate the expression of the target genes of rice *OsMYB4* and *OsWRKY45*, respectively, provided protection against the necrotrophic fungus *Cochliobolus miyabeanus* while increasing susceptibility to *M. oryzae* and *Xanthomonas oryzae* pv. *oryzae* (*Xoo*) [53]. Pathogen effectors target the *S* genes (susceptibility genes) of the host. In our work, the *S. nodorum* SnTox1 effector targets the wheat *S* gene *TaSnn1*. *S* genes are a promising new direction of research, since they determine the outcome of the disease and can modulate the main processes in plants; in some cases, *S* genes regulate the effect of ambivalence and can influence resistance/susceptibility through the effect on SA-JA/ethylene antagonism [3]. The effector of the causative agent of downy mildew Arabidopsis, *Hyaloperonospora arabidopsidis* (*Hpa*) HaRxL44, specifically interacts with Arabidopsis MEDIATOR COMPLEX SUBUNIT 19a (MED19a), resulting in a shift in the defense balance between the SA and JA/ethylene pathways, the repression of *PR1* gene expression, and the promotion of the growth of biotrophs (such as *Hpa*) through the suppression of the SA response. However, it does provide immunity against necrotrophs through an enhanced JA/ethylene response [69]. The identification of effector–target interactions in the host and the determination of the role of these interactions in the SA-JA/ethylene crosstalk are thus necessary for fully understanding the interactions between plant susceptibility and pathogen virulence, as well as is the further use of this knowledge to develop new strategies in breeding programs to overcome the ambivalence effect.

## 4. Materials and Methods

### 4.1. Plant and Fungi Materials and Growth Conditions

Six cultivars of bread spring and winter wheat (*Triticum aestivum* L.) were used in the work. Two cultivars (Chinese Spring (CS) and Mironovskaya808 (M808)) were historic wheat accessions from the N.I. Vavilov Institute of Plant Genetic Resources in Russia, one cultivar (Saratovskaya 29 (S29)) was obtained from the Federal Research Center Institute of Cytology and Genetics, the Siberian Branch of Russian Academy of Sciences (Novosibirsk, Russia), and three cultivars (Kazahstanskaya 10 (Kaz10), Omskaya 35 (Om35), and Zhnitsa (Zhn)) were modern commercial cultivars from the Institute of Biochemistry and Genetics Ufa Federal Research Center of the Russian Academy of Sciences (Ufa, Russia). All experimental plants were grown hydroponically on 10% Hoagland–Arnon nutrient medium for seven days under controlled conditions in a KBW E6 plant growth chamber (Binder GmbH, Tuttlingen, Germany) as described previously [18].

One isolate of the fungus *S. nodorum* Sn1SP (from the collection of Institute of Biochemistry and Genetics, Ufa Federal Research Centre, Russian Academy of Sciences, Ufa, Russia), which expressed the SnTox1 gene, was used in the work [18]. The *S. nodorum* Sn1SP isolate in the collection was maintained by cultivation on barley grains and maintained at 4 °C. To obtain the fungal spore preparation, barley grains with sporo-mycelial mass were soaked in sterile distilled water. Then, a suspension at a concentration of 10^6^ spores mL^−1^ was used to infect the plants.

### 4.2. Experimental Design

Experiments to assess the resistance/susceptibility of six wheat cultivars were carried out on the separated first leaves of 7-day-old seedlings placed in Petri dishes on wet cotton wool containing 0.004% benzamidazole (12–14 leaves/dish) [13]. Experiments to study biochemical characteristics and the expression of hormonal signaling pathways genes were performed on intact 7-day-old seedlings. In the case of studying the role of SA or JA, pre-sowing treatment was carried out by soaking the seeds for 3 h in a 50 μM SA solution (Merck KGaA, Sigma-Aldrich, Darmstadt, Germany) or in a 0.1 μM JA solution (Merck KGaA, Sigma-Aldrich, Darmstadt, Germany). The plants were then grown under controlled conditions in a KBW E6 plant growth chamber (Binder GmbH, Tuttlingen, Germany) as described previously [18]. The biologically effective concentration of SA and JA was selected according to [70,71,72,73]. In the case of studying the role of ethylene, intact 7-days-old seedlings placed in separate vessels and separated first leaves placed in Petri dishes were sprayed with 1.5 mM solution of 2-chloroethylphosphonic acid (ethephone, ET) (Merck KGaA, Sigma-Aldrich, Darmstadt, Germany) 24 h before infection with the pathogen *S. nodorum*, and then the vessels were closed with caps. The vessels and Petri dishes were then transferred into the darkness [74]. The solution of ET contained the wetting agent Tween-20 (0.02%). Control plants were sprayed with a solution containing only the wetting agent Tween-20 (0.02%). Inoculation of intact seedlings and separated leaves with a suspension of spores of the fungus *S. nodorum*, observation of the development of symptoms and fixation and measurement of damage zones were performed as described previously [13,18].

To study biochemical parameters or gene expression, shoots of intact wheat seedlings subjected to various treatments were fixed in liquid nitrogen 24 and 72 h after inoculation with the virulent *S. nodorum* isolate Sn1SP. To analyze *SnTox1* gene transcription during infection of different wheat genotypes, leaves of different wheat cultivars inoculated with *S. nodorum* isolate Sn1SP were collected 24 and 72 hpi.

### 4.3. Analysis of H_2_O_2_ Content and Redox Enzyme Activity

To measure H_2_O_2_ production and activity of redox enzymes (peroxidase (PO) and catalase (CAT)), plant material (1:5 weight/volume) was homogenized in 0.05 M solution of Na–phosphate buffer (PB), pH 6.2, and incubated at 4 °C for 30 min. Supernatants were separated by centrifugation at 15,000× *g* for 15 min (5415 K Eppendorf, Hamburg, Germany). The H_2_O_2_ concentration in the supernatant was determined by xylenol orange, in the presence of Fe^2+^, where hydroperoxides were reduced by ferrous ions in acid solution forming a ferric product–xylenol orange complex, detected spectrophotometrically at 560 nm [75]. PO activity was determined by the oxidation of (*o-*) phenylenediamine in the presence of 25 μL of 0.0016% H_2_O_2_ solution, as described previously [76]. Optical density at 490 nm was measured on a Benchmark Microplate Reader spectrophotometer (Bio-Rad Laboratories, Hercules, CA, USA) [77]. Enzymatic activity was expressed as optical density/mg protein per minute. CAT activity was determined using the standard method based on the ability of H_2_O_2_ to form a stable-colored complex with molybdate salts, as described previously [76]. Optical density was measured at 405 nm on a Benchmark Microplate Reader spectrophotometer (Bio-Rad Laboratories, Hercules, CA, USA). CAT activity was calculated using a calibration curve and expressed in µM H_2_O_2_/(mg protein per min). Protein content was determined by the Bradford method.

### 4.4. Gene Expression Analysis

Total wheat RNA and total fungal RNA were extracted using Lira^®^ (Biolabmix, Moscow, Russia) according to the manufacturer’s instructions. For cDNA synthesis, the method described in an earlier work was used [78]. Primers for real-time polymerase chain reaction (real-time PCR) were devised using the web tool PrimerQuest™ (https://www.idtdna.com/pages/tools/primerquest, accessed on 1 June 2024) (Integrated DNA Technologies, Inc., Coralville, IA, USA). The sequences of all the primers are presented in Table S2 (Supplementary Materials) for genes encoding enzymes of phytohormones biosynthesis, the TF of these hormonal signaling pathways and PR proteins. The annealing temperature of the primers was 60 °C. A melting curve analysis was conducted to determine the specificity of the reaction (at 95 °C for 15 s, 60 °C for 1 min and 95 °C for 15 s). The efficiency of the primers was determined using a series of cDNA dilutions (10-fold). To normalize the results of the expression of the studied wheat genes, primers to the genes of the constitutively expressed proteins RNase L inhibitor-like (*TaRLI*) and glyceraldehyde 3-phosphate dehydrogenase (*TaGAPDH*) were used as an internal control (Table S2 Supplementary Materials). To normalize the results ofthe expression of the fungal NE gene *SnTox1*, primers to the constitutively expressed *Snβ-tubulin* gene of *S. nodorum* were used (Table S2 Supplementary Materials). Real-time PCR was performed on a “DNA amplifier in real time” CFX96 Touch with fluorescent detection (BioRad Laboratories, Hercules, CA, USA). For detection, a set of reagents, EvaGreen I (Synthol, Moscow, Russia), was used. In order to quantify the relative gene expression, the 2^−ΔΔCT^ method was performed as described earlier [79]. Three independent biological and five technical replications were performed for each experiment.

### 4.5. Statistical Analysis

All experiments were repeated three times with three biological repetitions. Experimental data were expressed as means ± SE, which were calculated in all treatments using Microsoft Excel. The significance of differences was assessed by ANOVA followed by Duncan’s test (*p* ≤ 0.05) with STATISTICA 10.0 software (version STA999K347150-W, Tulsa, OK, USA). Before statistical analysis, the normal distribution of all parameters was analyzed and confirmed by the Shapiro–Wilk W test. The calculation of the Pearson’s correlation coefficients and the construction of correlation matrices were carried out using Microsoft Excel (version 16.0.14430.20306, Redmond, WA, USA). The treatment variants and the number of repetitions are indicated in the tables and figures.

## 5. Conclusions

**Figure 8:**
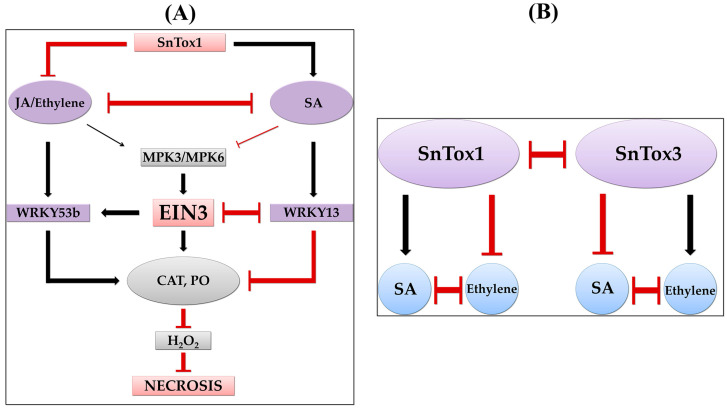
Model illustrating how NEs can manipulate host-plant hormonal signaling pathways. (**A**) SnTox1 uses antagonism of SA and JA/ethylene signaling pathways to produce necrosis. (**B**) Possible mechanism of epistasis between two NEs SnTox1 and SnTox3. Black lines with arrows represent stimulatory effects, and blunt red lines represent inhibitory effects.

Taken together, our data of the gene expression and redox status of resistant and sensitive wheat genotypes treated with SA, JA and ET showed that SnTox1 uses the ambivalence effect (antagonism between the SA and JA/ethylene signaling pathways) to regulate plant redox status and necrosis formation (Figure 8).

The results of the work showed that the SA signaling pathway is involved in the development of susceptibility, and the JA/ethylene signaling pathway is involved in the development of the resistance of wheat plants to the SnTox1-producing isolate of *S. nodorum* in the presence of a compatible SnTox1-*Snn1* interaction. NE SnTox1 hijacks the SA signaling pathway and uses it to suppress CAT activity, increases H_2_O_2_ content and induces necrosis formation; simultaneously, SnTox1 suppresses the JA and ethylene hormonal pathways by SA. To manipulate the antagonism of the SA and JA/ethylene signaling pathways, NE SnTox1 reprograms the expression of the MAPK genes *TaMRK3* and *TaMRK6* and the TF genes *TaWRKY13*, *TaEIN3* and *TaWRKY53b*. A schematic representation of these data is presented in Figure 8A.

It is known that SnTox1-*Snn1* and SnTox3-*Snn3-B1* interactions are epistatic to each other, but the mechanism of epistasis has not been fully elucidated [19,26]. Haugrud et al. (2019) hypothesized that the putative PTI pathway activated by a compatible SnTox1-*Snn1* interaction may antagonize or inhibit the response of the ETI pathways activated by other interactions such as SnTox3-*Snn3-B1* [19]. Based on the results of this work and our previous studies [13], a possible mechanism of epistasis between two NEs SnTox1 and SnTox3 is revealed, which may be associated with the hijack and manipulation by NE of plant hormonal pathways. Thus, SnTox1 hijacks the SA pathway and suppresses the ethylene signaling pathway, and SnTox3 hijacks the ethylene signaling pathway and suppresses the SA pathway for the formation of necrosis and pathogen development. A schematic representation of these data is presented in Figure 8B. Thus, our study provides new data on the role of the NE SnTox1 in manipulating hormonal pathways for the development of susceptibility on the role of SA, JA and ethylene in the development of the resistance/susceptibility of wheat to *S. nodorum*.

Thus, further research should focus on exploring the strategies by which pathogens deceive host plants within hormonal pathways. Future research should focus on further elucidating the molecular mechanisms underlying *S*-gene-modulated resistance and the loss of the function of *S* genes. Such approaches include the identification of partial mutants of the *S* gene or a strategy to control *S* gene transcription and translation [3]. This will require the use of hormonal treatments or plant hormonal mutants and transgenic plants or genome-edited plants. Deepening our knowledge in this area will be instrumental in developing new strategies in breeding programs and will contribute to the development of environmentally friendly sustainable agriculture.

## Figures and Tables

**Table 1:** The effect of treatment with phytohormones on the lesion area on leaves of six cultivars of varying degrees of sensitivity to NE SnTox1 infected with the *S. nodorum* Sn1SP.

Variant of Treatment	Cultivars					
	CS *	Om35	M808	Zhn	S29	Kaz10
Sn1SP	53.6 ± 5.1 f	50.3 ± 5.2 f	5.2 ± 0.6 b	2.3 ± 0.2 a	10.8 ± 1.4 c	11.2 ± 1.1 c
Sn1SP + SA	53.5 ± 4.9 f	63.7 ± 7.1 g	12.7 ± 1.5 c	31.7 ± 3.7 e	24 ± 3.1 de	18.5 ± 2.5 d
Sn1SP + JA	11.4 ± 2.3 c	19.9 ± 2.1 d	5.6 ± 0.7 b	2.4 ± 0.2 a	7.4 ± 0.9 bc	4.9 ± 0.5 b
Sn1SP + ET	29 ± 3.5 e	27.7 ± 3.4 e	9.4 ± 1.2 c	15.7 ± 1.5 cd	18.1 ± 2.2 d	12 ± 1.6 c

* Leaf area and lesion area were measured 6 days after infection with the pathogen isolate. The area of the damage zones is presented as a percentage of the total leaf area, which is taken as 100%. SA—salicylic acid, JA—jasmonic acid, ET—ethephon, a chemical precursor of ethylene. Values are expressed as mean SE (*n* = 30) (ANOVA with Duncan’s test; comparisons between genotypes are presented; significant differences are marked with different letters at *p* ≤ 0.05).

## Data Availability

Data are available in a publicly accessible repository.

## Supplementary Materials

The following supporting information can be downloaded at https://www.mdpi.com/article/10.3390/plants13182546/s1. Table S1: Regression statistics for six wheat cultivars treated with SA, JA and ET in which the expression of the *SnTox1* gene and damage zones was analyzed during infection with the Sn1SP isolate; Figure S1: The expression of three genes of enzymes for the biosynthesis of phytohormones—the SA (*TaPAL*) (**A**), the JA (*TaLOX*) (**B**) and the ethylene (*TaACS1*) (**C**)—and the expression of the *PR*–genes—*TaPR1* (**D**), *TaPR3* (**E**) and *TaPR6* (**F**) in six bread wheat cultivars 24 and 72 hpi with the SnTox1-producing isolate *S. nodorum* Sn1SP. Designations of wheat cultivars as in Figure 1. Average gene expression was calculated from nine biological samples in five technical replicates (*n* = 9). Values are expressed as mean SE (ANOVA with Duncan’s test; comparisons between genotypes are presented; significant differences are marked with different letters at *p* ≤ 0.05); Figure S2: The expression of six TF genes—*TaWRKY13* (**A**), *TaWRKY45* (**B**), *TaMYC2* (**C**), *TaEIN3* (**D**), *TaERF1* (**E**) and *TaWRKY53b* (**F**) in six bread wheat cultivars 24 and 72 hpi with the SnTox1-producing isolate *S. nodorum* Sn1SP. Designations of wheat cultivars as in Figure 1. Average gene expression was calculated from nine biological samples in five technical replicates (*n* = 9). Values are expressed as mean SE (ANOVA with Duncan’s test; comparisons between genotypes are presented; significant differences are marked with different letters at *p* ≤ 0.05); Table S2: The sequences of primers used for real-time PCR.
