# Supplementary material for: The Role of Salicylic, Jasmonic Acid and Ethylene in the Development of the Resistance/Susceptibility of Wheat to the SnTox1-Producing Isolate of the Pathogenic Fungus Stagonospora nodorum (Berk.)
Source: Plants (Basel). 2024 Sep 10;13(18):2546. doi: 10.3390/plants13182546 (PMC11435178; doi:10.3390/plants13182546)
Supplement: Supplementary file 1 [file plants-13-02546-s001.zip › Veselova et al-Suplimentry-Plants-2024.pdf]

plants

Article

The Role of Salicylic, Jasmonic Acid and Ethylene in the Development of Resistance/Susceptibility of Wheat to the SnTox1-producing Isolate of the Pathogenic Fungus *Stagonospora nodorum* (Berk.)

Svetlana Veselova [1,*], Tatyana Nuzhnaya [1,2], Igor Maksimov [1]

[1] Institute of Biochemistry and Genetics, Ufa Federal Research Centre, Russian Academy of Sciences, Prospekt Oktyabrya, 71, 450054 Ufa, Russia; tanyawww89@mail.ru (T.V.N.); maksimov@ufaras.ru (I.V.M.)
[2] Ufa Institute of Biology, Ufa Federal Research Centre, Russian Academy of Sciences, Prospekt Oktyabrya, 69, 450054 Ufa, Russia
* Correspondence: veselova75@rambler.ru; Tel.: +7-9173423941

plants

MDPI

Supplementary Table S1. Regression statistics for six wheat cultivars treated with SA, JA and ET, in which the expression of the *SnTox1* gene and damage zones was analyzed during infection with the Sn1SP isolate.

Regression Statistics	
Multiple R	0.885182
R Square	0.78354718
Adjusted R Square	0.77370842
Standard Error	0.94960811
Observations	24

ANOVA

	df	*SS*	*MS*	*F*	*Significance F*
Regression	1	71.8147109	71.8147109	79.6387778	9.18088E-09
Residual	22	19.8386224	0.90175556		
Total	23	91.6533333			

	Coefficients	*Standard Error*	*t Stat*	*P-value*	*Lower 95%*	*Upper 95%*	*Lower 95.0%*	*Upper 95.0%*
Intercept	0.41001702	0.3025721	1.3551052	0.18913581	-0.217479103	1.03751315	-0.2174791	1.03751315
X Variable 1	0.09932054	0.01112953	8.92405613	9.1809E-09	0.076239311	0.122401772	0.076239311	0.122401772

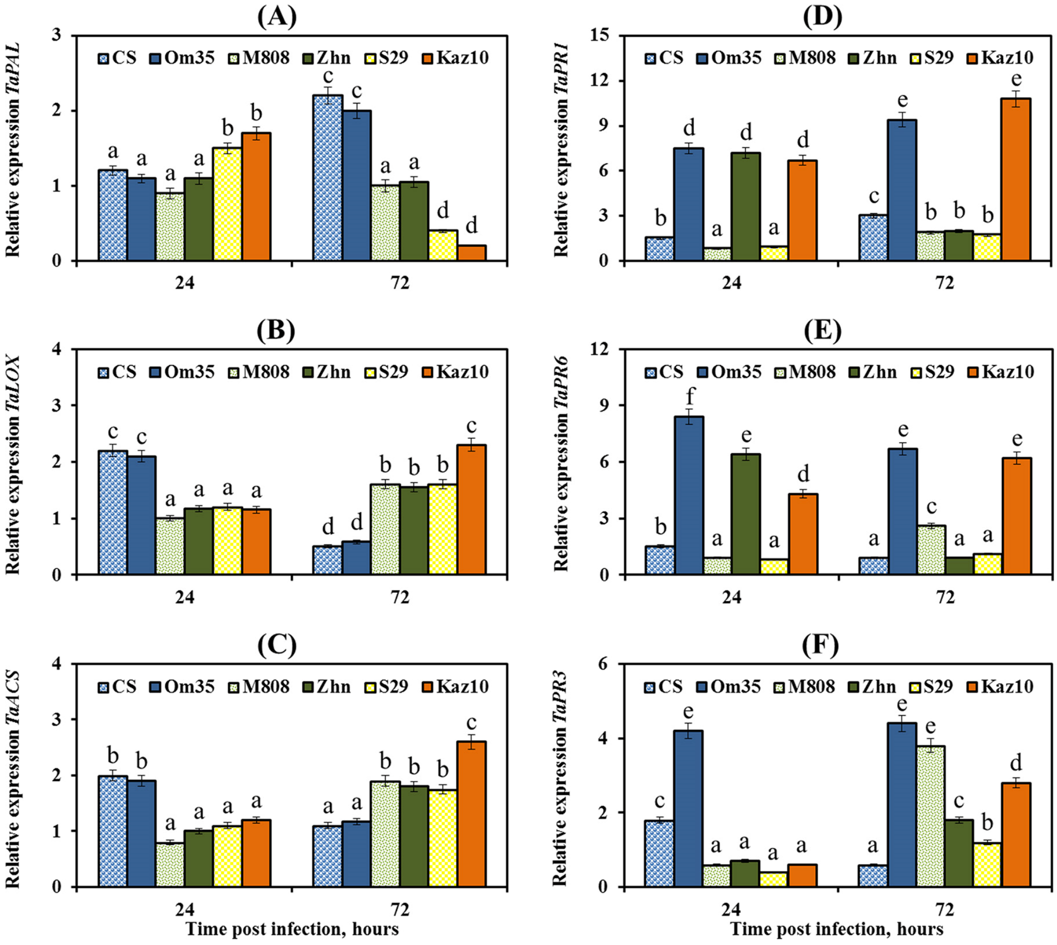

Supplementary Figure S1. The expression of three genes of enzymes for the biosynthesis of phytohormones – the SA (*TaPAL*) (**A**), the JA (*TaLOX*) (**B**), and the ethylene (*TaACS1*) (**C**), and the expression of the *PR*–genes – *TaPR1* (**D**), *TaPR3* (**E**), and *TaPR6* (**F**) in six bread wheat cultivars 24 and 72 hpi with the SnTox1-producing isolate *S. nodorum* Sn1SP. Designations of wheat cultivars as in Figure 1. Average gene expression was calculated from nine biological samples in five technical replicates (n = 9). Values are expressed as mean SE (ANOVA with Duncan's test; comparisons between genotypes are presented; significant differences are marked with different letters at $p \leq 0.05$).

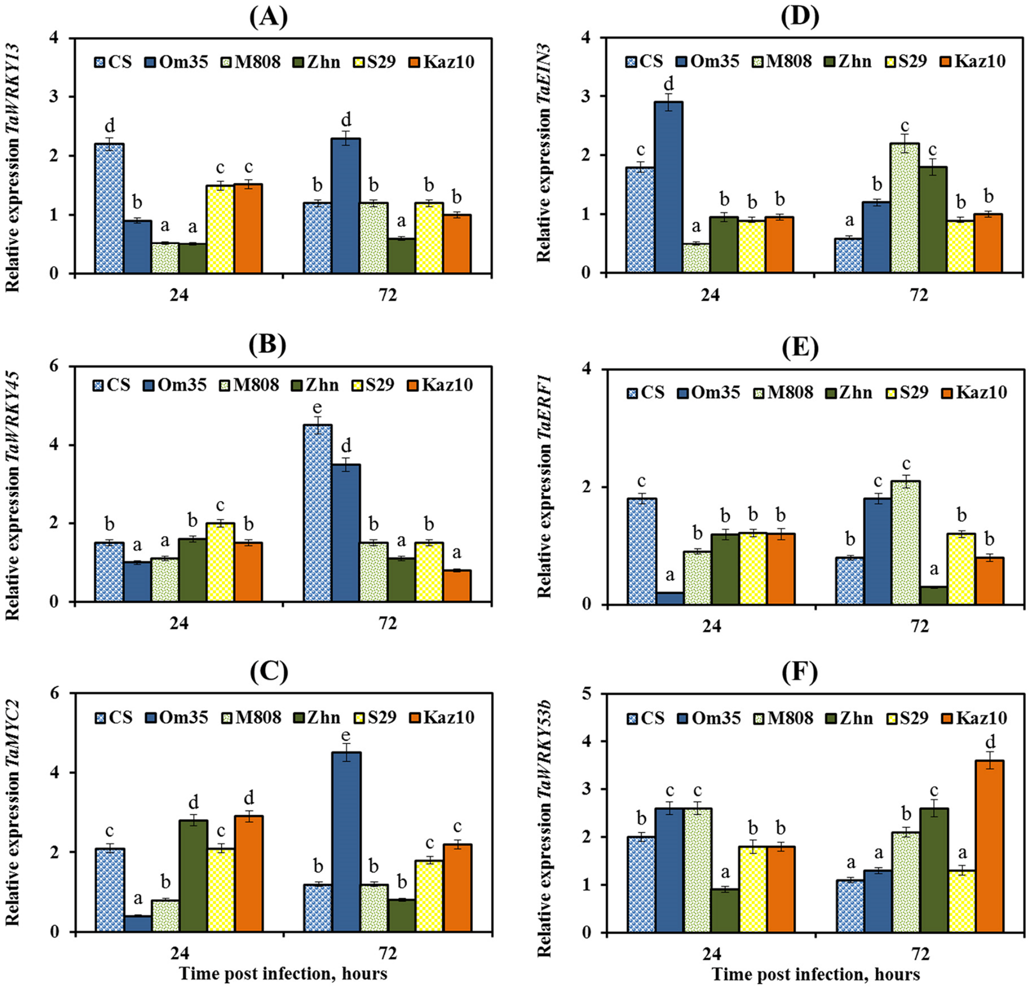

Supplementary Figure S2. The expression of six TFs genes – *TaWRKY13* (**A**), *TaWRKY45* (**B**), *TaMYC2* (**C**), *TaEIN3* (**D**), *TaERF1* (**E**) and *TaWRKY53b* (**F**) in six bread wheat cultivars 24 and 72 hpi with the SnTox1-producing isolate *S. nodorum* Sn1SP. Designations of wheat cultivars as in Figure 1. Average gene expression was calculated from nine biological samples in five technical replicates (n = 9). Values are expressed as mean SE (ANOVA with Duncan's test; comparisons between genotypes are presented; significant differences are marked with different letters at $p \leq 0.05$).

Supplementary Table S2. The sequences of primers used for real-time PCR.

Genes	Strand	5′ to 3′ Primer Sequences	GenBank accession number
TaPR1	Forward	ATAACCTCGGCGTCTTCATC	AF384143
	Reverse	GCTTATTACGGCATTCCTTTT	
TaPR3	Forward	CCATCCAGATCTCACACAACTAC	AB029936
	Reverse	ACCACAACGCCGTCTTAAA	
TaPR6	Forward	GGGCCCTGCAAGAAGTACTG	EU293132.1
	Reverse	ACACGCATAGGCACGATGAC	
TaWRKY13	Forward	GAGGAGGAACATGAGCAGAAG	EF397614
	Reverse	GAACCACCCGAAGTCGAAG	
TaWRKY45	Forward	ATTGCGCGTCGTAGGTTTAG	EF397613
	Reverse	GACGGATGGATGAGTAGCAAAG	
TaWRKY53b	Forward	CTCTCTGGCTTCTCCTTTCAC	EF368364
	Reverse	CTCCTGCTGCTCCTTGAATAA	
TaERF1	Forward	ACACATGCCGACGAATCTAC	EF583940
	Reverse	CCGAGTTCAAACCCAAATTCAC	
TaEIN3	Forward	GGCTCAACAACCTGGATTTT	KU030837
	Reverse	CCCTGTCCAAAGAAACCCT	
TaACS1	Forward	GAGGTTGTGAACGAGCTAGAAG	U35779
	Reverse	CTCGAAACCCTGCAAGAGATAG	
TaPAL	Forward	GGCGTCAAAACATGGCGTC	X99725
	Reverse	AGTCCGAGAAGTCCGAGA	
TaLOX	Forward	AGTCCGAGAAAACATGGCGTC	BJ223744
	Reverse	ATAGTCCGAGACTCCAAG	
TaMYC2	Forward	AGTCTGGTGTCCTGGTTACA	AY625684.1
	Reverse	GGTGTTGTCCCTTCCTCTTTAC	
TaMPK3	Forward	TACATGAGGCACCTGCCGCAGT	AF079318
	Reverse	GGTTCAACTCCAGGGCTTCGTTG	
TaMPK6	Forward	GAAGATATCCGCCAACTTCCCCG	AY173962
	Reverse	CGCATGCTGCTCGAAGTCAAAGC	
SnTox1	Forward	GTACTCCCGTACGTACTCTTCT	JX997402
	Reverse	CGCTTGTTTGCCGTTCTTAC	
TaGAPDH	Forward	GTGTTCCCACTGTTGATGTTTC	EU022331.1
	Reverse	CCTCCTTGATAGCAGCCTTAAT	
Snβ-tubulin	Forward	ACACCAGGAACAACTGCTAACAGC	S56922
	Reverse	TATGCGCGCGTGCTGCAAATTCGA	
TaRLI	Forward	TTGAGCAACTCATGGACCAG	AY059462
	Reverse	GCTTTCCAAGGCACAAACAT	
